# Clinical Roles of Risk Model Based on Differentially Expressed Genes in Mesenchymal Stem Cells in Prognosis and Immunity of Non-small Cell Lung Cancer

**DOI:** 10.3389/fgene.2022.823075

**Published:** 2022-02-24

**Authors:** Qiang Guo, Xiao-Yue Xiao, Chuang-Yan Wu, Dan Li, Jiu-Ling Chen, Xiang-Chao Ding, Chao Cheng, Chong-Rui Chen, Song Tong, Si-Hua Wang

**Affiliations:** ^1^ Department of Thoracic Surgery, Union Hospital, Tongji Medical College, Huazhong University of Science and Technology, Wuhan, China; ^2^ Department of Cardiovascular Surgery, Union Hospital, Tongji Medical College, Huazhong University of Science and Technology, Wuhan, China; ^3^ Department of Oncology, Huanggang Central Hospital, Huanggang, China; ^4^ Department of Thoracic Surgery, Renmin Hospital of Wuhan University, Wuhan, China; ^5^ Department of Thoracic Surgery, West China Hospital, Sichuan University, Chengdu, China

**Keywords:** NSCLC, MSCs, postn, TRPA1, DDIT4, nomogram, risk model

## Abstract

The tumor microenvironment (TME) plays an important regulatory role in the progression of non-small cell lung cancer (NSCLC). Mesenchymal stem cells (MSCs) in the TME might contribute to the occurrence and development of cancer. This study evaluates the role of differentially expressed genes (DEGs) of MSCs and the development of NSCLC and develops a prognostic risk model to assess the therapeutic responses. The DEGs in MSCs from lung tissues and from normal tissues were analyzed using GEO2R. The functions and mechanisms of the DEGs were analyzed using the Gene ontology (GO) and Kyoto Encyclopedia of Genes and Genomes (KEGG). Additionally, the Cancer Genome Atlas (TCGA) database was used to determine the expression levels of the DEGs of MSCs in the NSCLC tissues. The prognostic factors of NSCLC related to MSCs were screened by survival analysis, meta-analysis, Cox regression analysis, and a prognostic risk model and nomogram was developed. The signaling mechanisms and immune roles that risk model participate in NSCLC development were determined via Gene Set Enrichment Analysis and CIBERSORT analysis. Compared to the normal tissues, 161 DEGs were identified in the MSCs of the lung tissues. These DEGs were associated with mechanisms, such as DNA replication, nuclear division, and homologous recombination. The overexpression of *DDIT4*, *IL6*, *ITGA11*, *MME*, *MSX2*, *POSTN*, and *TRPA1* were associated with dismal prognosis of NSCLC patients. A high-risk score based on the prognostic risk model indicated the dismal prognosis of NSCLC patients. The nomogram showed that the age, clinical stage, and risk score affected the prognosis of NSCLC patients. Further, the high-risk model was associated with signaling mechanisms, such as the ECM-receptor interaction pathways, cytokine-cytokine receptor interaction, and MAPK pathways, involved in the progression of NSCLC and was also related to the components of the immune system, such as macrophages M0, T follicular helper cells, regulatory T cells. Therefore, the risk model and nomogram that was constructed on the basis of MSC-related factors such as *POSTN*, *TRPA1*, and *DDIT4* could facilitate the discovery of target molecules that participate in the progression of NSCLC, which might also serve as new candidate markers for evaluating the prognosis of NSCLC patients.

## Introduction

The tumor microenvironment (TME) is composed of mesenchymal stem cells (MSCs), immune cells, blood vessels, fibroblasts, smooth muscle cells, epithelial cells, and the extracellular matrix. It plays an important regulatory role in the progression of non-small-cell lung cancer (NSCLC) (Lee and Cheah, 2019; Horvath et al., 2020; Jia M. et al., 2021). For example, the CD133^+^ cell subtype CD2 cells of lung adenocarcinoma (LUAD) and NSCLC patients are related to malignancy and have stem cell-like characteristics. CD2^+^ cells promote tumor growth in nude mice. CD133 cells regulate the conversion of CD133^+^ subgroup cells into CD2^+^ Th17-like cells to promote the growth of lung cancer (Jia M. et al., 2021). Low levels of IFNγ in the TME increase the risk of tumor metastasis during immunotherapy, indicating that IFNγ induces cancer progression (Song et al., 2019).

In recent years, risk models, nomograms, and immunological assays have become tools for evaluating the prognosis of cancer patients and for discovering new mechanisms that could delay cancer progression (Hu et al., 2020; Guo et al., 2021; Chao et al., 2021; Wang L. et al., 2021; Zhang et al., 2021). Melatonin reduces the viability and induces apoptosis of mutant*-KRAS* NSCLC cells. Programmed death ligand 1 (PD-L1) is overexpressed in mutant-*KRAS* NSCLC cells. Melatonin treatment downregulates the expression of PD-L1 and effectively inhibits the expression of *YAP* and *TAZ*. Further, melatonin downregulates the expression of genes downstream of YAP/TAZ. PD-L1 expression is positively correlated with *YAP* and *TAZ* in NSCLC patients. The overexpression of PD-L1 indicates poor-survival outcomes in cancer patients. Thus, the downregulated expression of PD-L1 in *KRAS*-mutant NSCLC due to melatonin regulates tumor immunity (Chao et al., 2021).

In the TME, MSCs participate in material transportation and intercellular information transmission by secreting exosomes, and regulate tumor cell viability (Liu et al., 2021; Mohr et al., 2021; Wang W. et al., 2021; Muralikumar et al., 2021; Yin et al., 2021). Co-culturing MSCs with pancreatic cancer cells shows enhanced expression of metastatic cytokines CCL2 and IL6, which are produced by the stem cells. The Fas/FasL signaling pathway in MSCs is related to the progression and metastasis of pancreatic cancer (Mohr et al., 2021). Long non-coding RNA (lncRNA) DNM3OS is upregulated in hepatocellular carcinoma (HCC). The overexpression of DNM3OS is associated with the TNM stage, vascular invasion, and dismal prognosis in HCC patients. DNM3OS enhances HCC cell proliferation, invasion, and metastasis, as well as tumor occurrence and metastasis, *in vivo*. Tumor MSCs can promote HCC proliferation, invasion, and metastasis through the DNM3OS/KDM6B/TIAM1 signaling axis. In addition, MSCs induce an increase in the expression of DNM3OS in HCC cells (Wang W. et al., 2021). The expression of miR-204 is decreased in NSCLC tissues. Exosomes of MSCs promote the migration and invasion of NSCLC A549 cells, and enhance the expression of epithelial-mesenchymal transition (EMT) proteins, such as E-cadherin, N-cadherin, Vimentin, KLF7, *p*-AKT/AKT, and HIF-1α. Overexpression of miR-204 in the exosomes of MSCs inhibits KLF7 expression and AKT/HIF-1α pathway activity, leading to cell migration and invasion (Liu et al., 2021).

MSCs play an important role in the progression of NSCLC (Liu et al., 2021). However, the mechanisms by which MSCs contribute to the progression of NSCLC have not yet been fully elucidated. Therefore, we analyzed differentially expressed genes (DEGs) in the MSCs of lung tissues to elucidate the biological functions and signaling pathways involved in the progression of NSCLC. We obtained data regarding the DEGs in the MSCs of lung tissues from the GSE104636 dataset of Gene Expression Omnibus (GEO) database using the R package, GEO2R (Fregni et al., 2018). A nomogram, risk model, and network of the hub DEGs of MSCs were constructed by identifying and screening NSCLC data provided in The Cancer Genome Atlas (TCGA) and Lung Cancer Explorer (LCE) databases to provide new candidate molecules and targets for NSCLC treatment.

## Materials and Methods

### DEGs in MSCs of Lung Cancer Tissues

The GSE104636 dataset was obtained from the GEO database.^1^ The GSE104636 dataset comprises a microarray analysis of gene expression data of MSCs of cancer tissues and adjacent normal tissues in nine lung patients (Fregni et al., 2018). The information of the GSE104636 dataset is provided under the platform ID GPL6244 [HuGene-1_0-st] Affymetrix Human Gene 1.0 ST Array (transcript [gene] version). In the present study, GEO2R analysis was used to investigate the DEGs in the MSCs of normal tissues and of lung tissues. As described in a previous study, the absolute value of logFold change (FC) > 0.585 and *p* < 0.05 were defined as the criteria for significant difference (Zhang et al., 2021).

### Biological Functions and Signaling Mechanisms of DEGs

Gene ontology (GO) annotation and Kyoto Encyclopedia of Genes and Genomes (KEGG) analysis were used to analyze the biological functions and signaling mechanisms associated with the DEGs (Guo et al., 2020). An adjusted-P value <0.05 was considered statistically significant.

### Protein-Protein Interaction Network of DEGs

A PPI network was used to show the potential interactions between different proteins. MSC-related DEGs were entered into the Search Tool for the Retrieval of Interacting Genes (STRING) database^2^ to obtain the PPI network and explore their roles using the MCODE plug-in of the Cytoscape (version 3.8.2) software.

### Identification of Hub DEGs

Gene expression data and clinical data of NSCLC, available on the official website of TCGA^3^ as of August 2021, were downloaded. The data for DEGs of MSCs in NSCLC tissues were extracted from the downloaded data, and the limma R/Bioconductor software package was used to identify whether the genes of MSCs were abnormally expressed in NSCLC tissues, and the absolute value of logFC is greater than 1 and *p* < 0.05 as the research screening criteria. The potential effects of DEGs of MSCs on the overall survival (OS) of the NSCLC patients were determined by Kaplan-Meier (K-M) survival analysis with *p* < 0.05 as the filter condition.

### Prognostic Nomogram of Factors Associated With DEGs

The lung Cancer Explorer (LCE) database^4^ is a bioinformatics reanalysis database mainly based on the data from the TCGA and GEO databases. The LCE database features an analysis of gene expression levels for the prognosis of lung cancer, LUAD, and LUSC patients. In this study, the values of prognostic DEGs of MSCs related to the prognosis of lung cancer were identified via meta-analysis, with *p* < 0.05 considered statistically significant, and a prognostic nomogram of the DEGs of MSCs was constructed.

### Risk Model Based on Factors Associated With DEGs

Univariate Cox regression analysis was used to investigate the relationship between the expression levels of DEGs of MSCs and the prognosis of NSCLC patients, and *p* < 0.05 was used as the filter criterion. Multivariate Cox regression analysis and the Akaike information criterion (AIC) were used to evaluate the relationship between the levels of DEGs and the prognosis of NSCLC patients, and risk score for the NSCLC patients in our study was assigned accordingly. The relationship between the risk model constructed on the basis of the factors associated with the prognostic DEGs and the survival time and clinicopathological characteristics of NSCLC patients are showed using the heatmap and K-M survival analysis. The effect of risk score on the prognosis of NSCLC patients is shown using univariate Cox regression analysis and a nomogram that was verified by receiver operating characteristic (ROC) analysis. Visualization of the relationship between DDIT4, POSTN and TRPA1 and risk models using correlation analysis.

### Consensus Clustering

The NSCLC data from the TCGA database was divided into two groups according to the POSTN, TRPA1 and DDIT4 gene expression levels using the R Consensus ClusterPlus package, followed by principal component analysis (PCA). Survival rates of the two cluster groups of NSCLC patients were clustered by using K-M survival analysis.

### Gene Set Enrichment Analysis

GSEA was used to explore the possible functions and mechanisms associated with each gene. The risk scores of NSCLC patients were ranked, and the gene expression data of NSCLC patients obtained from TCGA were grouped according to the median risk value to explore the signaling pathways enriched in the high-risk and low-risk groups (Zhang et al., 2021). Signaling pathways with nominal (NOM) *p* < 0.05 was regarded as significantly enriched in the two risk groups.

### Analysis of Immune Cells

The expression levels of 22 immune cells in each of the 1,037 samples of NSCLC (data obtained from TCGA) were analyzed using the CIBERSORT method. The expression levels of immune cells and cell markers and risk scores were combined and sorted, which divided into two groups based on median value of risk score. The expression levels of immune cells and immune cell markers in the two groups were investigated using the *t*-test.

### Statistical Analysis

Data obtained from the TCGA and GEO databases were processed and visualized using programs created in Perl and R. The prognosis-related DEGs of MSCs in NSCLC patients were assessed using survival analysis, Cox regression analysis, and the nomogram. Univariate Cox regression, multivariate Cox regression, meta-analysis, and AIC were used to construct a risk model for NSCLC patients. *p* < 0.05 was considered statistically significant.

## Results

### DEGs in MSCs of Lung Cancer Tissues

**FIGURE 1:**
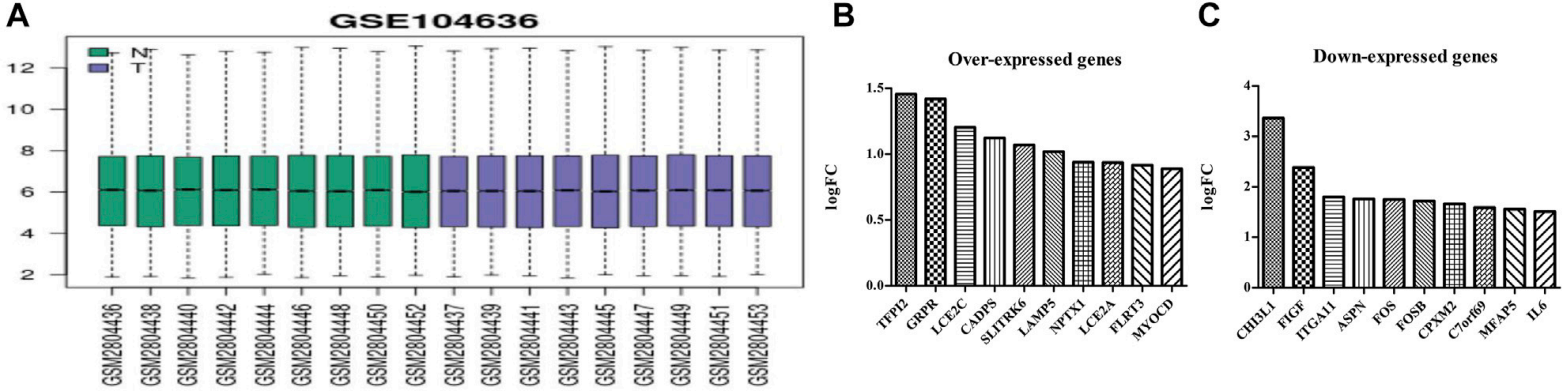
Sample quality and gene expression in lung cancer tissues of the GSE104636 dataset. **(A)** Sample quality; **(B)** Overexpressed genes; **(C)** Under-expressed genes.

The GSE104636 dataset had good quality samples of lung cancer and normal tissues (Figure 1A). GEO2R analysis showed 161 DEGs in the MSCs of lung cancer samples, and the differential expression was statistically significant (Supplementary Table S1). The top 10 overexpressed genes in MSCs of lung cancer tissues were as follows: *SLITRK6*, *MYOCD*, *GRPR*, *LCE2C*, *CADPS*, *TFPI2*, *LAMP5*, *NPTX1*, *LCE2A*, and *FLRT3* (Figure 1B). The top 10 under-expressed genes were as follows: *CHI3L1*, *FIGF*, *ITGA11*, *ASPN*, *FOS*, *FOSB*, *CPXM2*, *C7orf69*, *MFAP5*, and *IL6* (Figure 1C).

### Functional Mechanisms and Network of DEGs of MSCs

**FIGURE 2:**
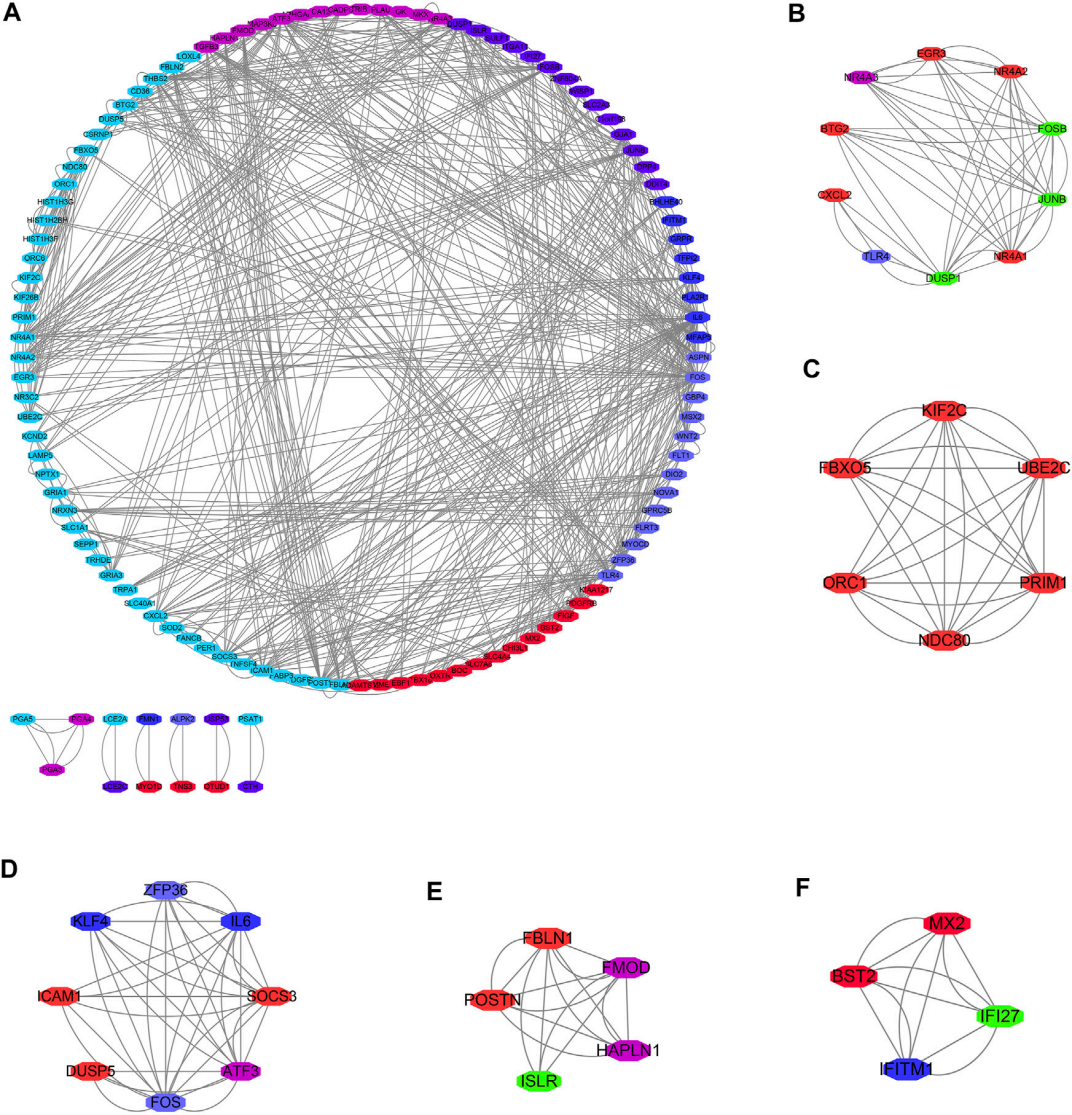
PPI network of DEGs of MSCs. **(A)** PPI network; **(B–F)** Cluster of PPI network obtained via the MCODE plug-in of Cytoscape. PPI, Protein-protein interaction; MSCs, Mesenchymal stem cells; DEGs, differentially expressed genes.

GO annotation results showed that the DEGs were significantly enriched in DNA replication, chromosome segregation, nuclear division, sister chromatid segregation, regulation of mitotic cell cycle phase transition, G2/M transition of mitotic cell cycle, DNA conformation change, regulation of DNA metabolic process, and other functions (Supplementary Table S2). KEGG pathway analysis showed that the DEGs were significantly enriched in cell cycle, RNA transport, P53 signaling pathway, DNA replication, spliceosome, homologous recombination, cellular senescence, and other signaling mechanisms (Table 1), and the results are presented as a bubble diagram (Supplementary Figure S1). In addition, Figure 2 shows the constructed PPI network of DEGs of MSCs.

**TABLE 1:** Signaling mechanisms of DEGs of MSCs determined via KEGG analysis.

ID	Description	P	FDR
hsa04110	Cell cycle	3.98E-31	7.40E-29
hsa03030	DNA replication	6.77E-26	6.30E-24
hsa03040	Spliceosome	9.13E-21	5.66E-19
hsa03460	Fanconi anemia pathway	6.37E-14	2.96E-12
hsa03440	Homologous recombination	2.21E-10	8.22E-09
hsa03430	Mismatch repair	5.70E-09	1.77E-07
hsa03050	Proteasome	1.01E-08	2.69E-07
hsa03410	Base excision repair	2.67E-08	6.21E-07
hsa04114	Oocyte meiosis	9.79E-08	2.02E-06
hsa03420	Nucleotide excision repair	1.72E-07	3.21E-06
hsa03013	RNA transport	1.82E-06	3.07E-05
hsa03008	Ribosome biogenesis in eukaryotes	3.01E-06	4.66E-05
hsa04914	Progesterone-mediated oocyte maturation	5.81E-06	8.31E-05
hsa00670	One carbon pool by folate	6.89E-06	9.15E-05
hsa04218	Cellular senescence	5.46E-05	0.000677153
hsa05166	Human T-cell leukemia virus 1 infection	0.00013996	0.001627031
hsa03015	mRNA surveillance pathway	0.00084735	0.009271006
hsa04115	p53 signaling pathway	0.001957051	0.020222864

Note: DEGs, differentially expressed genes; MSCs, Mesenchymal stem cells; KEGG, kyoto encyclopedia of genes and genomes.

### Screening of MSC-Associated Factors Related to the Prognosis of NSCLC Patients

**FIGURE 3:**
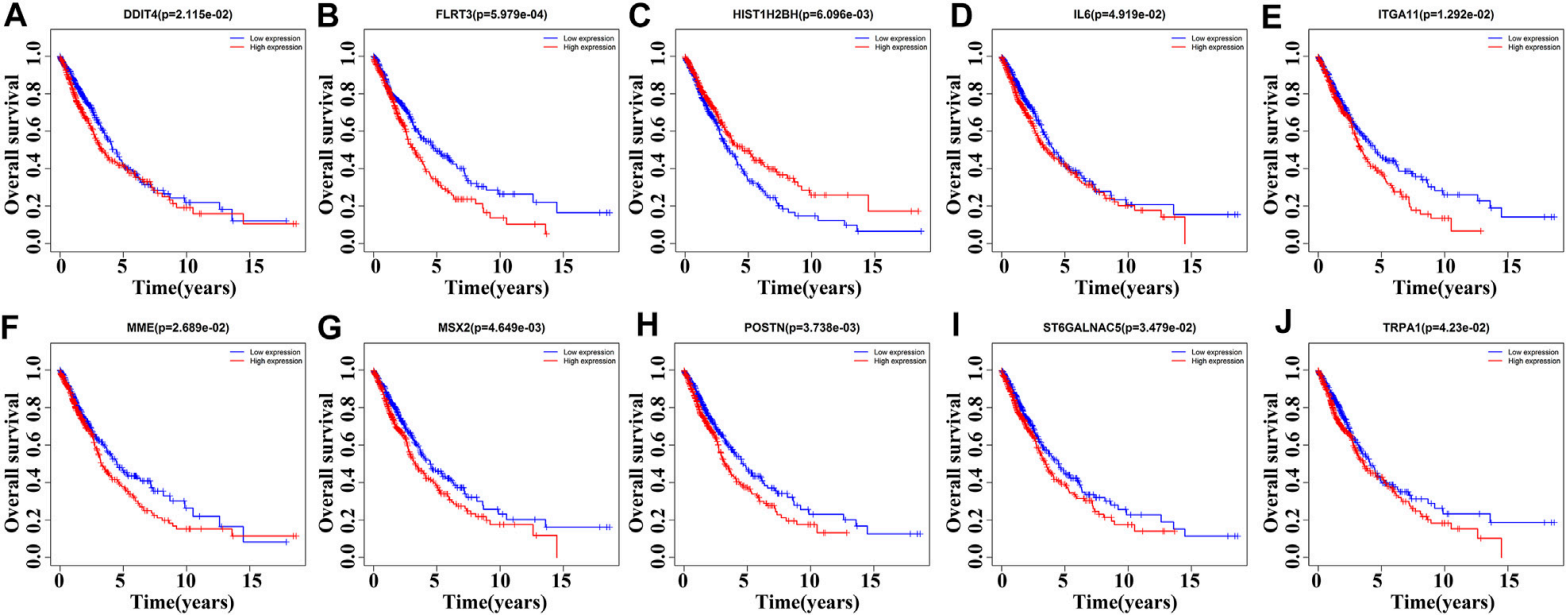
Roles of DEGs of MSCs in the overall survival of NSCLC patients determined via survival analysis. **(A)** *DDIT4*; **(B)** *FLRT3*; **(C)** *HIST1H2BH*; **(D)** *IL6*; **(E)** *ITGA11*; **(F)** *MME*; **(G)** *MSX2*; **(H)** *POSTN*; **(I)** *ST6GALNAC5*; **(J)** *TRPA1*. MSCs, Mesenchymal stem cells; DEGs, differentially expressed genes; NSCLC, Non-small-cell lung cancer.

85 DEGs were present in NSCLC tissues of TCGA database using our screening criteria (Supplementary Figure S2 and Table 2). K-M survival analysis revealed that the expression levels of *HIST1H2BH, IL6, MME, ST6GALNAC5*, *DDIT4*, *FLRT3*, *ITGA11*, *MSX2*, *POSTN*, and *TRPA1* were associated with the dismal prognosis of NSCLC patients (Figure 3). Among them, the overexpression of *IL6, MME, ST6GALNAC5*, *DDIT4*, *FLRT3*, *ITGA11*, *MSX2*, *POSTN*, and *TRPA1* and low expression of *HIST1H2BH* were associated with the dismal prognosis of NSCLC patients.

**TABLE 2:** The DEGs of MSCs in NSCLC tissues (TCGA database).

Gene	logFC	*p* Value	Gene	logFC	*p* Value	Gene	logFC	*p* Value
ITGA11	2.607	7.75E-36	PRIM1	1.283	5.24E-34	IL6	−2.694	4.27E-22
KIF26B	2.713	1.77E-50	TBX18	2.819	1.52E-10	EGR3	−1.561	9.40E-18
DDIT4	1.485	1.72E-20	SULF2	1.420	4.49E-12	GBP4	−1.315	1.27E-35
POSTN	1.672	2.82E-22	PLA2R1	1.136	1.98E-05	FBXO5	1.341	6.76E-39
SLC1A1	−2.467	1.94E-57	ARRB1	−1.690	5.88E-54	TENM3	1.225	5.10E-05
NDC80	3.661	1.20E-60	FOSB	−3.444	5.17E-42	UBE2C	4.674	6.34E-62
CXCL2	−2.840	2.86E-38	PER1	−1.358	5.46E-36	PSAT1	4.428	2.08E-58
SHISA3	1.159	1.47E-28	CA12	2.577	1.62E-11	NR3C2	−1.343	1.11E-41
SLIT3	−2.595	4.50E-59	TNFSF4	1.288	4.22E-19	CRABP2	5.068	6.78E-54
CD36	−2.801	7.58E-60	FANCB	2.575	5.08E-52	ORC6	3.745	1.20E-62
SOCS3	−1.542	7.44E-20	FLRT3	−1.951	6.07E-53	TRHDE	−2.792	5.52E-57
LAMP5	1.635	2.15E-17	WNT2	−1.047	3.49E-31	GRIA1	−3.942	1.95E-61
CLIC2	−1.109	2.39E-38	OLFML1	−1.695	2.47E-52	SULF1	2.348	5.48E-40
ALPK2	2.752	2.32E-38	TRPA1	4.219	8.45E-20	THBS2	2.540	8.04E-42
MT1M	−3.009	1.28E-46	KIF2C	4.092	9.03E-63	EBF1	-1.036	1.81E-34
C1orf198	−1.279	4.06E-51	ATF3	−1.641	3.14E-27	HIST1H2BH	5.258	1.97E-39
SLC12A8	1.711	5.84E-45	ZFP36	−1.959	6.04E-40	HIST1H3F	6.763	1.32E-28
NR4A2	−1.257	2.16E-20	PALMD	−1.730	8.86E-51	MAP3K8	−1.180	5.63E-35
PLAU	2.801	2.74E-42	FOS	−1.876	6.21E-33	NR4A1	−2.106	4.16E-38
KLF4	−1.544	5.55E-39	DUSP1	−1.893	2.13E-48	TSHZ2	1.012	1.19E-07
JUNB	−1.001	4.15E-18	MME	−1.598	8.77E-47	GCNT4	−1.068	2.26E-35
CSRNP1	−2.631	3.58E-55	OTUD1	−1.792	1.08E-59	TNS3	−1.227	9.71E-47
DIO2	3.039	1.62E-38	MYOCD	−2.216	2.41E-50	CHI3L1	1.152	0.00011984
TLR4	−1.642	4.66E-53	ORC1	3.355	5.42E-60	ST6GALNAC5	−2.144	1.05E-55
MSX2	1.891	2.77E-12	ICAM1	−1.435	4.05E-29	ADAMTS12	1.800	2.52E-26
NRK	3.314	3.93E-14	WISP1	2.126	5.20E-35	HIST1H3G	4.253	2.42E-32
ZNF724	2.120	2.10E-31	CEMIP	1.684	1.13E-16	PDGFRL	1.173	4.31E-13
KCND2	2.337	1.36E-26	NR4A3	−2.486	7.83E-37	LCE2C	4.058	0.000419976
BTG2	−1.250	1.39E-35						

Note: DEGs, differentially expressed genes; MSCs, Mesenchymal stem cells; NSCLC, non-small cell lung cancer; TCGA, the cancer genome atlas.

### Construction of the Prognostic Nomogram of DEGs of MSCs

**FIGURE 4:**
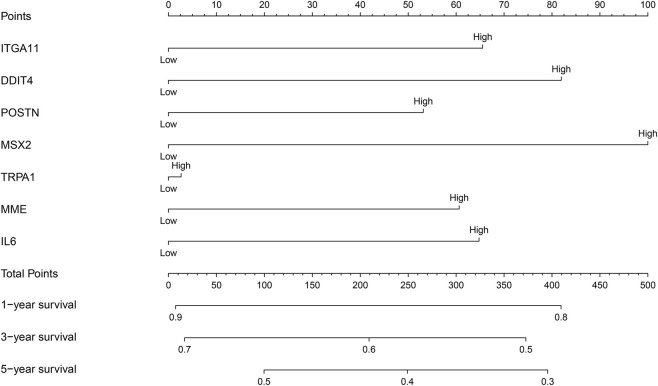
Nomogram of prognostic DEGs of MSCs. MSCs, Mesenchymal stem cells; DEGs, differentially expressed genes.

The relationship between the expression levels of *HIST1H2BH, IL6, MME, ST6GALNAC5*, *DDIT4*, *FLRT3*, *ITGA11*, *MSX2*, *POSTN*, and *TRPA1* and the prognosis of NSCLC patients were examined by meta-analysis of data obtained from the LCE database. The results showed that the overexpression of *DDIT4, IL6, ITGA11*, *MME, MSX2*, *POSTN*, and *TRPA1* were strongly associated with the dismal prognosis of NSCLC patients, and were statistically significant (Supplementary Figure S3). Therefore, a prognostic nomogram of these prognosis related DEGs was constructed by grouping the median values of the expression levels of *DDIT4, IL6, ITGA11*, *MME, MSX2*, *POSTN*, and *TRPA1* (Figure 4). In the nomogram of MSCs-related genes, MSX2 had the greatest impact on the prognosis of NSCLC patients, followed by DDIT4, while TRPA1 had the least impact on the prognosis of NSCLC patients.

### Construction of the Risk Model and Nomogram

**FIGURE 5:**
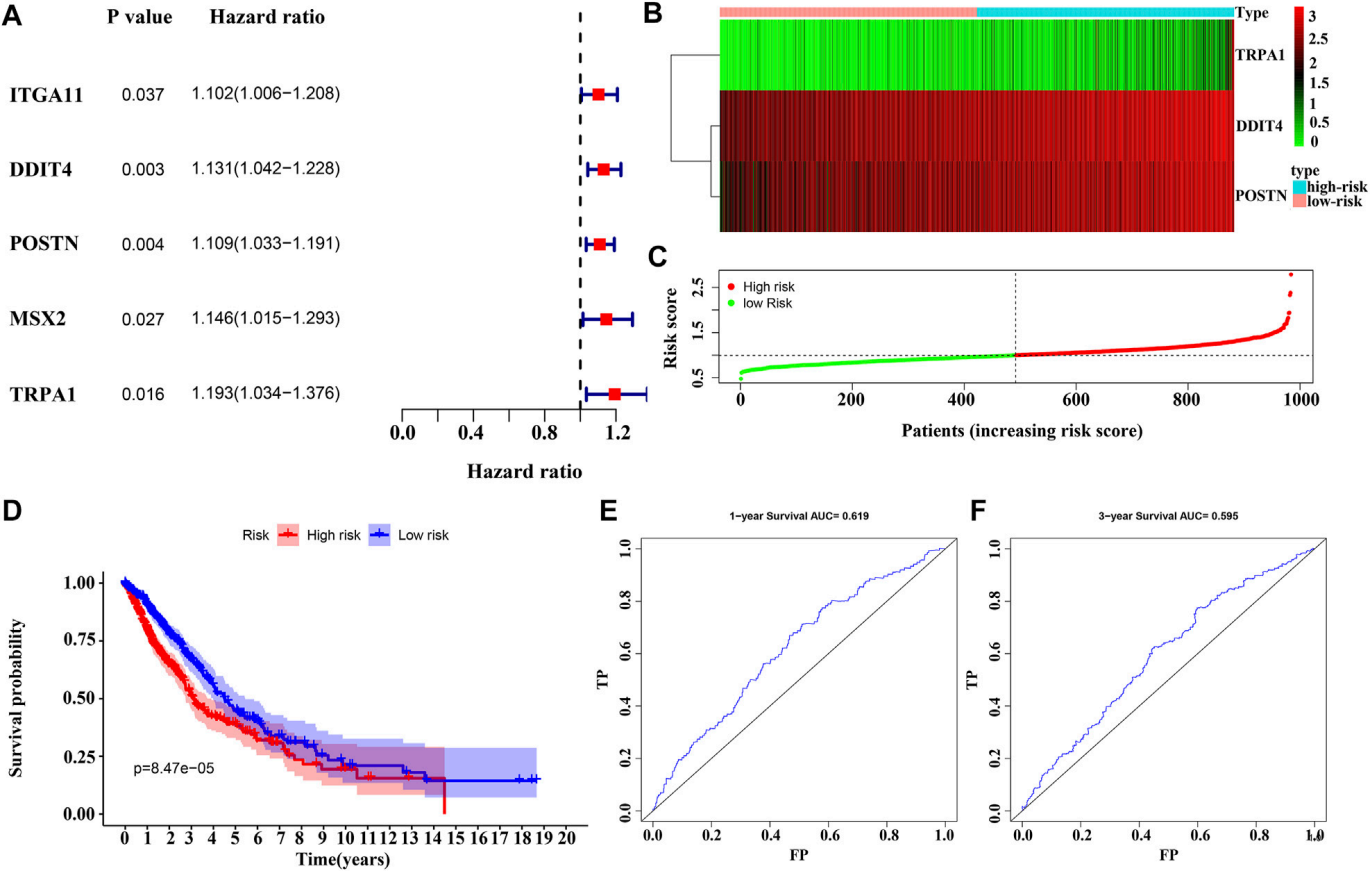
Construction of MSC DEGs prognostic risk model. **(A–B)** Prognostic model factors of NSCLC patients using COX analysis; **(C–D)** Risk score correlates with the survival time of NSCLC patients; **(E–F)** The usefulness of risk model using the ROC analysis. NSCLC, non-small-cell lung cancer; ROC, receiver operating characteristic; MSCs, Mesenchymal stem cells; DEGs, differentially expressed genes.

Univariate Cox regression analysis revealed that the overexpression of *ITGA11*, *POSTN*, *DDIT4*, *MSX2*, and *TRPA1* were risk factors for the OS of NSCLC patients (Figure 5A). Overexpression of *POSTN, TRPA1* and *DDIT4* independently influenced the dismal prognosis of NSCLC patients, according to the results of the multivariate Cox regression analysis and AIC (Figure 5B). Figures 5C,D show the relationship between the risk score and the prognosis of NSCLC patients according to the risk model constructed in this study, and have appraised value using ROC analysis (Figures 5E,F).

**FIGURE 6:**
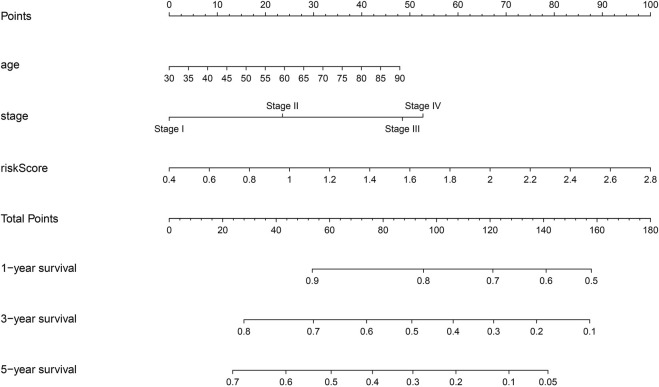
Construction of nomogram for risk model.

The prognostic genes *POSTN, TRPA1*, and *DDIT4* showed high correlation with the risk model using correlation analysis and represented as a heatmap (Supplementary Figures S4, S5A). Risk scores correlating with the gender, clinical stage, lymph node metastasis, T stage, M stage, and survival time are represented via heatmap (Supplementary Figure S5A). Risk score, clinical stage, lymph node metastasis, age, and T stage were associated with dismal prognosis of NSCLC patients according to the univariate Cox regression analysis (Supplementary Figures S5B). Risk score, clinical stage, and age were associated with dismal prognosis of NSCLC patients according to the multivariate Cox regression analysis (Supplementary Figures S5C). The nomogram of the risk score, clinical stage, age, and dismal prognosis of NSCLC patients was constructed which suggests that the risk score affected the prognosis of NSCLC patients more than the clinical stage (Figure 6).

### Consensus Clustering of Risk Model Genes Identifies Distinct Clinical Outcomes in NSCLC Patients

**FIGURE 7:**
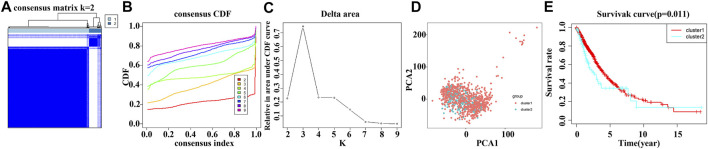
Consensus clustering of risk model genes identifies dismal prognosis in NSCLC patients. **(A–C)** Consensus clustering of risk model genes; **(D)** Significant differences between the NSCLC patients of Cluster1 and Cluster2 groups using PCA analysis. **(E)** Survival time of NSCLC patients in Cluster1 and Cluster2 groups using survival analysis. NSCLC, non-small-cell lung cancer; PCA, principal component analysis.

Consensus clustering analysis of the TCGA NSCLC data for risk model genes *POSTN*, *TRPA1* and *DDIT4* showed that k = 2 was the best grouping (Figures 7A–C). Consensus clustering analysis was performed with k = 2 and grouped into Cluster1 and Cluster2 groups with significant differences. PCA analysis showed that there was a significant difference between the Cluster1 and Cluster2 groups based on the data of NSCLC patients in the TCGA database (Figure 7D). K-M survival analysis showed that the survival time of NSCLC patients in the Cluster1 group was significantly higher than that of the NSCLC patients in the Cluster2 group (Figure 7E).

### Determination of Signaling Mechanisms Associated With the Risk Model

GSEA analysis revealed that the high-risk model was associated with the pathways in cancer, ECM receptor interaction, P53 signaling pathway, NOD like receptor signaling pathway, cytokine-cytokine receptor interaction, MAPK signaling pathway, leukocyte transendothelial migration, Toll like receptor signaling pathway, natural killer cell mediated cytotoxicity and other signaling pathways involved in the progress of NSCLC (Supplementary Figure S6 and Table 3).

**TABLE 3:** Signaling mechanisms associated with the risk model.

Name	Size	NES	NOM p
ECM receptor interaction	84	2.3963685	0
Focal adhesion	199	2.2862277	0
Small cell lung cancer	84	2.0498757	0
Glycosaminoglycan biosynthesis chondroitin sulfate	22	2.0387504	0.001872659
P53 signaling pathway	68	2.0353227	0.002004008
Regulation of actin cytoskeleton	212	1.99435	0
Pathways in cancer	325	1.9349892	0
Arrhythmogenic right ventricular cardiomyopathy arvc	74	1.9192715	0.001848429
NOD like receptor signaling pathway	62	1.8920375	0.002024292
Cytokine-cytokine receptor interaction	263	1.8466959	0.003992016
Axon guidance	129	1.8341341	0.007619048
Melanoma	71	1.795697	0.003968254
Hypertrophic cardiomyopathy hcm	83	1.7617054	0.011695907
Dilated cardiomyopathy	90	1.7332542	0.005905512
Glioma	65	1.7241526	0.003984064
Bladder cancer	42	1.6860583	0.01002004
Renal cell carcinoma	70	1.6804767	0.01369863
Pathogenic escherichia coli infection	55	1.6623056	0.015444015
Leukocyte transendothelial migration	116	1.6476305	0.034026466
Natural killer cell mediated cytotoxicity	131	1.6152565	0.04117647
GAP junction	89	1.6110837	0.018255578
Prion diseases	35	1.6003833	0.018
TOLL like receptor signaling pathway	102	1.5973029	0.034951456
Pancreatic cancer	70	1.5835325	0.019646365
Glycosaminoglycan biosynthesis heparan sulfate	26	1.5168692	0.04696673
MAPK signaling pathway	267	1.494283	0.017374517

Note: NES, normalized enrichment score; NOM, nominal.

### Associations of the Risk Model With the NSCLC TME

**FIGURE 8:**
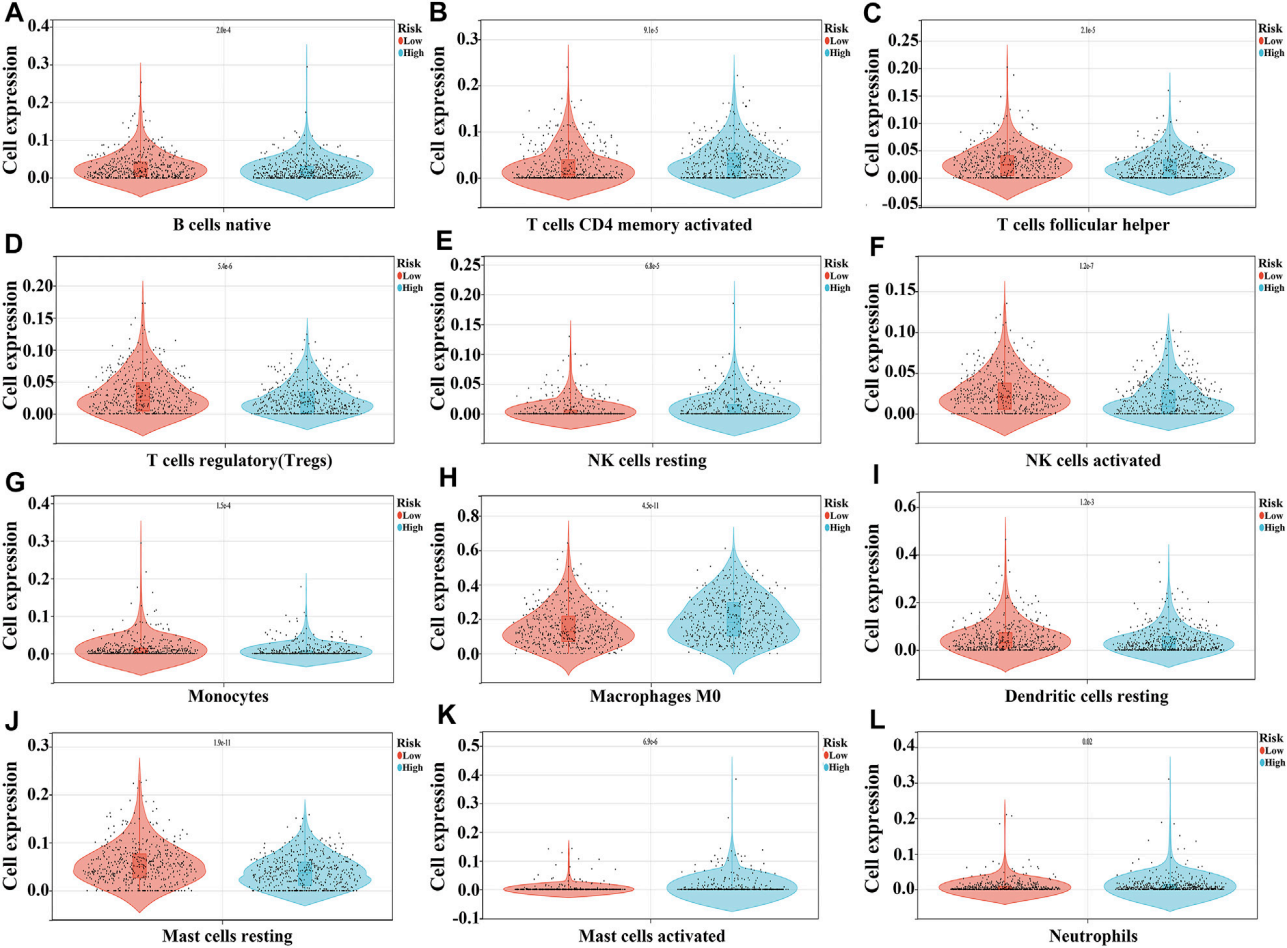
The expression levels of NSCLC immune cells in the high- and low-risk groups. **(A)** B cells naïve; **(B)** T cells CD4 memory activated; **(C)** T cells follicular helper; **(D)** T cells regulatory; **(E)** NK cells resting; **(F)** NK cells activated; **(G)** Monocytes; **(H)** Macrophages M0; **(I)** Dendritic cells resting; **(J)** Mast cells resting; **(K)** Mast cells activated; **(L)** Neutrophils.

In the high-risk group, the expression of macrophages M0, mast cells activated, NK cells resting, neutrophils, and B cells memory were significantly increased, and the expression of mast cells resting, NK cells activated, T cells regulatory (Tregs), T cells follicular helper, T cells CD4 memory activated, monocytes, B cells naïve, and dendritic cells resting were extremely decreased, as compared to the expression levels in the low-risk group (Figure 8 and Table 4).

**TABLE 4:** The expression levels of NSCLC immune cells in the high- and low-risk groups.

Immune cells	Low-risk	High-risk	P
B cells naive	0.03 ± 0.03	0.02 ± 0.03	2.00E-04
B cells memory	7.7e-3 ± 0.02	5.1e-3 ± 0.02	0.04
Plasma cells	0.10 ± 0.09	0.10 ± 0.08	0.67
T cells CD8	0.09 ± 0.07	0.08 ± 0.07	0.1
T cells CD4 naive	1.8e-4 ± 2.4e-3	1.1e-4 ± 1.3e-3	0.57
T cells CD4 memory resting	0.12 ± 0.08	0.12 ± 0.08	0.9
T cells CD4 memory activated	0.02 ± 0.04	0.03 ± 0.04	9.10E-05
T cells follicular helper	0.03 ± 0.03	0.02 ± 0.02	2.10E-05
T cells regulatory	0.03 ± 0.03	0.02 ± 0.02	5.40E-06
T cells gamma delta	7.4e-3 ± 0.02	7.6e-3 ± 0.02	0.85
NK cells resting	6.8e-3 ± 0.02	0.01 ± 0.02	6.80E-05
NK cells activated	0.03 ± 0.02	0.02 ± 0.02	1.20E-07
Monocytes	0.01 ± 0.03	7.5e-3 ± 0.02	1.50E-04
Macrophages M0	0.15 ± 0.11	0.20 ± 0.13	4.50E-11
Macrophages M1	0.07 ± 0.05	0.08 ± 0.05	0.18
Macrophages M2	0.15 ± 0.07	0.15 ± 0.07	0.25
Dendritic cells resting	0.05 ± 0.06	0.04 ± 0.05	1.20E-03
Dendritic cells activated	0.02 ± 0.04	0.02 ± 0.04	0.29
Mast cells resting	0.06 ± 0.04	0.04 ± 0.03	1.90E-11
Mast cells activated	3.5e-3 ± 0.02	0.01 ± 0.03	6.90E-06
Eosinophils	1.6e-3 ± 5.7e-3	1.5e-3 ± 8.5e-3	0.96
Neutrophils	8.6e-3 ± 0.02	0.01 ± 0.03	0.02

Note: NSCLC, non-small cell lung cancer.

**FIGURE 9:**
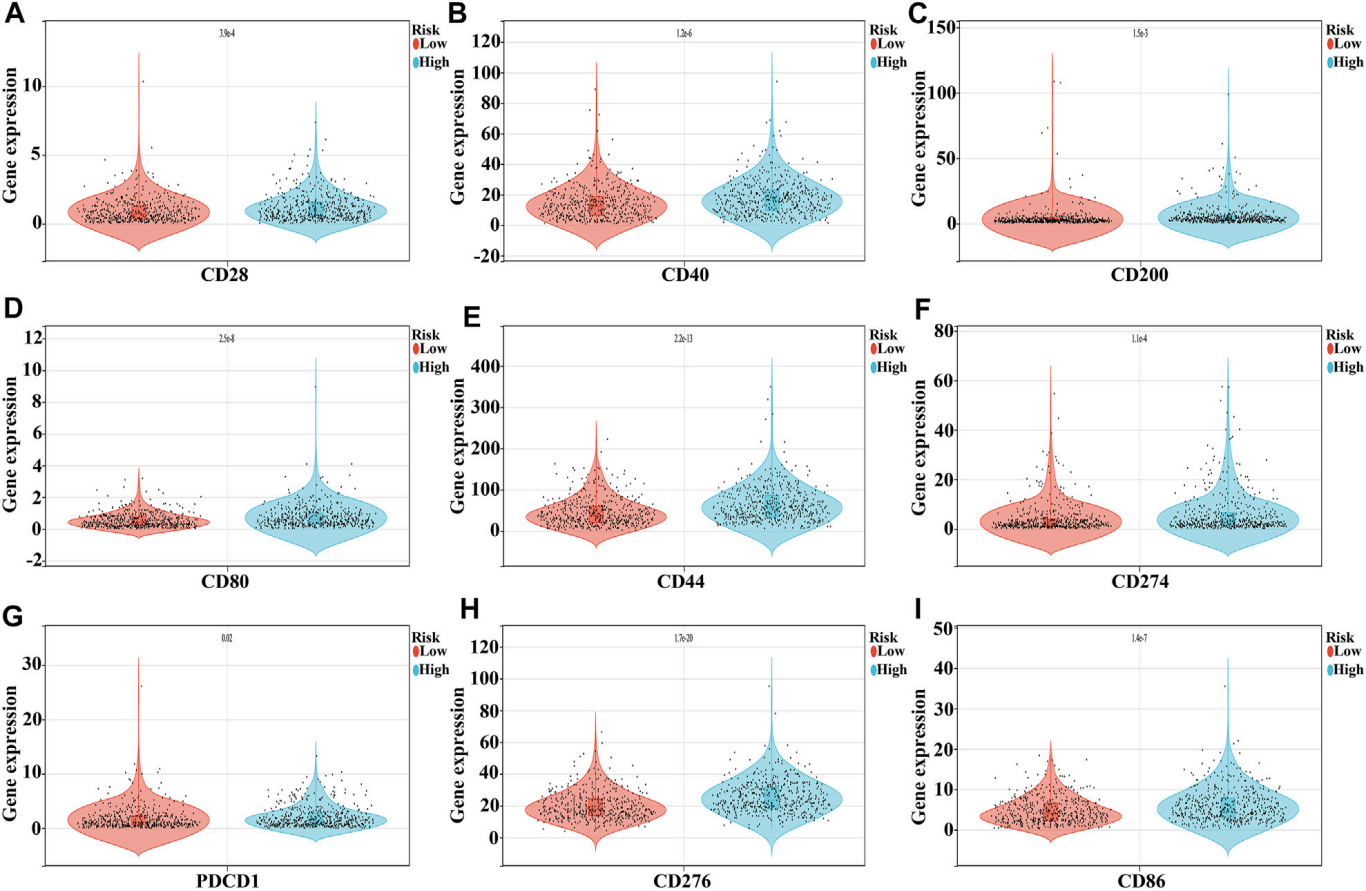
Expression levels of NSCLC immune cell markers in the high- and low-risk groups. **(A)** CD28; **(B)** CD40; **(C)** CD200; **(D)** CD80; **(E)** CD44; **(F)** CD274; **(G)** PDCD1; **(H)** CD276; **(I)** CD86.

Compared to the low-risk group, the expression of *CD276, TNFSF4, CD44, PDCD1LG2, CD80, CD86, VSIR, CD40, TNFRSF9, HAVCR2, CD274, ICOS, CTLA4, CD28, TNFRSF4, CD27, TIGIT, TMIGD2, CD200, CD70, TNFSF9, PDCD1, LAIR1,* and *TNFRSF14* were significantly increased, and the expression of *TNFSF15* and *CD40LG* were extremely decreased in the high-risk group (Figure 9 and Table 5).

**TABLE 5:** The expression levels of NSCLC immune cell markers in the high- and low-risk groups.

Immune genes	Low-risk	High-risk	P
CD160	0.18 ± 0.20	0.16 ± 0.17	0.07
KIR3DL1	0.05 ± 0.13	0.06 ± 0.26	0.32
HAVCR2	5.45 ± 4.28	6.70 ± 4.82	1.80E-05
TMIGD2	0.40 ± 0.45	0.51 ± 0.62	1.20E-03
PDCD1	1.75 ± 2.16	2.07 ± 2.03	0.02
TNFRSF25	4.69 ± 4.16	4.69 ± 4.06	0.98
TNFSF9	3.07 ± 2.69	3.61 ± 3.81	0.01
CD28	0.94 ± 0.91	1.15 ± 0.98	3.90E-04
TIGIT	1.41 ± 1.41	1.71 ± 1.47	1.00E-03
CTLA4	1.77 ± 2.08	2.26 ± 2.08	2.20E-04
TNFRSF18	9.84 ± 14.74	11.25 ± 10.28	0.08
CD44	45.53 ± 34.57	63.99 ± 42.79	2.20E-13
CD86	4.73 ± 3.33	5.98 ± 4.07	1.40E-07
TNFRSF4	2.77 ± 2.22	3.82 ± 6.35	5.30E-04
TNFSF18	0.89 ± 2.83	0.91 ± 2.14	0.93
TNFSF15	2.82 ± 3.82	1.48 ± 1.79	4.80E-12
NRP1	11.69 ± 9.52	11.41 ± 8.82	0.63
TNFSF4	0.99 ± 1.09	1.81 ± 1.66	4.80E-19
VTCN1	7.10 ± 15.09	7.14 ± 12.09	0.96
CD200R1	0.69 ± 0.76	0.67 ± 0.58	0.76
HHLA2	5.68 ± 17.58	4.29 ± 18.74	0.23
BTNL2	0.06 ± 0.11	0.06 ± 0.10	0.5
CD40	14.19 ± 10.95	17.72 ± 11.69	1.20E-06
CD200	4.17 ± 9.10	5.94 ± 8.43	1.50E-03
ICOSLG	0.12 ± 0.20	0.14 ± 0.23	0.29
CD80	0.55 ± 0.47	0.77 ± 0.72	2.50E-08
IDO2	0.16 ± 0.42	0.14 ± 0.48	0.46
BTLA	0.44 ± 0.56	0.47 ± 0.55	0.47
IDO1	13.53 ± 25.60	15.45 ± 26.42	0.25
LAG3	2.90 ± 3.66	3.27 ± 3.19	0.09
CD70	1.01 ± 4.79	2.16 ± 7.61	4.90E-03
TNFSF14	0.76 ± 0.84	0.72 ± 0.95	0.51
ICOS	0.96 ± 1.01	1.21 ± 1.07	2.00E-04
LAIR1	4.11 ± 3.45	4.55 ± 3.41	0.04
LGALS9	15.38 ± 13.38	14.01 ± 12.10	0.09
TNFRSF9	0.95 ± 1.18	1.44 ± 2.08	6.80E-06
CD276	19.72 ± 9.02	25.56 ± 10.25	1.70E-20
TNFRSF14	10.07 ± 8.41	9.15 ± 5.85	0.04
CD244	0.69 ± 0.79	0.71 ± 0.73	0.64
ADORA2A	0.09 ± 0.07	0.10 ± 0.08	0.26
CD27	6.40 ± 7.02	8.13 ± 8.95	7.80E-04
CD48	6.57 ± 9.00	7.26 ± 8.32	0.21
PDCD1LG2	2.16 ± 1.99	3.56 ± 4.11	2.10E-11
CD274	4.22 ± 6.14	6.03 ± 8.34	1.10E-04
VSIR	9.28 ± 5.29	11.11 ± 6.15	7.10E-07
CD40LG	0.89 ± 1.12	0.74 ± 0.87	0.02

Note: NSCLC, non-small cell lung cancer.

## Discussion

Several studies have confirmed the importance of the biological functions of MSCs in the progression of NSCLC (Fregni et al., 2018; Gu et al., 2020; Liang et al., 2020; Liu et al., 2021; Yin et al., 2021). MSCs are known to cause transcriptional changes in lung cancer cells, leading to increased expression of MMP9. High expression of MMP9 is related to reduced OS of LUAD patients. The involvement of MSCs in the progression of NSCLC is associated with ABL tyrosine kinase. MSCs can activate ABL tyrosine kinase in lung cancer cells. Functional ABL tyrosine kinase is a hub enzyme for MSCs that induces MMP9 expression (Gu et al., 2020). miR-144 showed significantly low expression in NSCLC tissues and cells, and high expression levels of CCNE1 and CCNE2 were observed. Overexpression of miR-144 can inhibit cell proliferation, colony formation, and S-phase arrest by downregulating CCNE1 and CCNE2 expression. Moreover, the proliferation and colony formation of NSCLC cells can be inhibited by miR-144 from the MSC-derived exosomes (Liang et al., 2020).

At present, the roles of MSCs in the progression of NSCLC have not been fully revealed. In this study, we found that HIST1H2BH, IL6, MME, ST6GALNAC5, DDIT4, FLRT3, ITGA11, MSX2, POSTN, and TRPA1 were abnormally expressed in NSCLC tissues. The overexpression of IL6, MME, ST6GALNAC5, DDIT4, FLRT3, ITGA11, MSX2, POSTN, and TRPA1, and low expression of HIST1H2BH was significantly related to the poor prognosis of NSCLC patients. Our risk model identified *POSTN, TRPA1* and *DDIT4* to be important factors affecting the prognosis of NSCLC patients. The expression of POSTN in pan-cancer and NSCLC tissues are significantly increased. POSTN is significantly expressed in renal cell carcinoma (RCC). Interference with the expression of POSTN can inhibit the proliferation, migration, and invasion of RCC cells, and inhibit EMT through the ILK/AKT/mTOR signaling pathway (Jia Y. Y. et al., 2021). The overexpression of POSTN is related to the clinical stage, degree of malignancy, lymph node metastasis, and OS in NSCLC patients, and it is also an independent risk factor for OS in NSCLC patients. POSTN can induce the expression of vimentin and N-cadherin, and downregulate the expression of E-cadherin, and promote the proliferation and migration of A549 cells (Hong et al., 2010; Ratajczak-Wielgomas et al., 2020). POSTN derived from cancer-associated fibroblasts (CAFs) is highly enriched in high-grade serous ovarian cancer (HGSC) stromal cells. POSTN overexpression is associated with reduced OS in patients with HGSC. POSTN derived from CAFs is a ligand for integrin αvβ3, which promotes the migration and invasion of ovarian cancer cells by activating the PI3K/AKT pathway and inducing EMT. TGF-β1 induces cancer metastasis and fibroblast activation, which is related to the expression of POSTN (Yue et al., 2021). Knockdown of TRPA1 promoted LLC-2 cell proliferation and invasiveness of lung cancer. TRPA1 induced autophagy under adverse conditions, and the combination of *TRPM8* and *TRPA1* directly contributed to the aggressive phenotype of lung cancer (Du et al., 2014). Methyl syringate inhibited hypoxia-induced COX-2 expression and promoter activity, reduced hypoxia-induced lung cancer cell migration and invasion, and inhibited vascular endothelial growth factor secretion (Park et al., 2016). TRPA1 antagonist antagonism could reverse cell migration and invasion. The expression level of DDIT4 in LUAD tissues was significantly higher than that in adjacent lung tissues. High DDIT4 expression level was associated with the shorter OS and was an independent predictor of OS of LUAD patients (Song et al., 2021). Blocking SIRT1/2 expression induced autophagy in NSCLC cells, increased the expression levels of ATF4 and DDIT4, and downregulated the levels of mTORC1 downstream molecules *p*-RPS6KB1 and *p*-EIF4EBP1. SIRT1/2 inhibition lead to acetylation of HSPA5, inducing the ER stress, and upregulation of ATF4 and DDIT4, triggering autophagy (Mu et al., 2019). This study gives a preliminarily demonstration that the screened MSC-related factors-*POSTN, TRPA1*, and *DDIT4*-have an important role in the progression of NSCLC.

P53 signaling pathway, DNA replication, ECM-receptor interaction, TGF-beta signaling pathway, chemokine signaling pathway, MAPK signaling pathway, cell cycle, and other signaling pathway are known to be involved in cancer (Constanzo et al., 2016; Zhan et al., 2017; Becker et al., 2019; Tsukita et al., 2019; Chi et al., 2020; Shen et al., 2020; Nozaki and Nishizuka, 2021). GINS2 is closely related to DNA replication and DNA damage, participates in cell cycle regulation, and is a hub protein in cell proliferation and apoptosis. GINS2 expression is significantly increased in NSCLC tissues and cells. Knockout of *GINS2* can inhibit cell proliferation and cause cell cycle arrest in G2/M phase. Knockdown of GINS2 expression can increase apoptosis and the expression of the apoptosis-related protein BAX, decrease the expression of the protein BCL-2, and induce the overexpression of P53 and GADD45A. Interference with GINS2 expression can downregulate cell proliferation through the P53/GADD45A signaling mechanism, induce cell cycle arrest in G2/M phase, and increase cell apoptosis (Chi et al., 2020). Interfering with RHOJ expression can promote TGF-β expression in A549 and PC-9 cells. RHOJ knockdown can enhance the invasion ability of A549 cells, and increase SMAD3 phosphorylation and SNAIL expression to regulate the EMT process (Nozaki and Nishizuka, 2021). In future studies, the roles and regulatory mechanisms of *POSTN, TRPA1,* and *DDIT4*, which are hub proteins of MSCs, in the progression of NSCLC should be confirmed, and new inhibitors should be developed to delay the progression of NSCLC and improve the prognosis of NSCLC patients.

TME dynamics are inseparable from the progression of NSCLC. MSCs are an important component of the TME (Zhao et al., 2018; Zheng et al., 2020; Yin et al., 2021). MSC-conditioned medium can promote EMT, invasion, and migration of lung cancer cells, and can inhibit cell proliferation and apoptosis. The EMT-promoting effect of MSCs is mediated by exosomes secreted by the MSCs and is eliminated by inhibiting the release of exosomes. Silencing the expression of TGF-β1 in MSCs by using MSCs exosomes can reverse the promotion of EMT and can enhance the anti-proliferation and pro-apoptosis effects of MSCs on lung cancer cells (Zhao et al., 2018). In our study, we elucidated that the risk model was related to the NSCLC microenvironment. Thus, in this study, the risk model constructed for MSCs in TME showed correlations involved in the progression of NSCLC.

Overall, the roles and mechanisms of the hub proteins of MSCs, *POSTN*, *TRPA1*, and *DDIT4*, were investigated via bioinformatic screening and analysis for their involvement in the progression of NSCLC in this study. The large sample size of the data involved in our research affirms the robustness of our analysis. Although, the TCGA and GEO databases contain sequencing data from many research centers, the risk model and nomogram from our study need to be confirmed using gene expression analysis in clinical samples. Along with this, the roles of MSC factors, *POSTN*, *TRPA1,* and *DDIT4* in mechanisms involved in the upstream and downstream processes which might influence NSCLC progression need to be confirmed by our team through basic research experiments such as immunoblotting, cell transfection, and transwell assay. Overexpression of MSC factors *DDIT4, IL6, ITGA11*, *MME, MSX2*, *POSTN*, and *TRPA1* were associated with dismal prognosis of NSCLC patients. Moreover, overexpression of *POSTN, TRPA1,* and *DDIT4* were independent factors influencing the dismal prognosis of NSCLC patients. A high-risk score based on our risk model is related to the dismal prognosis of NSCLC patients. The nomogram showed that the risk score, clinical stage, and age contributed to the dismal prognosis of NSCLC patients. The risk model was associated with ECM-receptor interaction, cytokine-cytokine receptor interaction, MAPK, and other signaling pathways involved in the progression of NSCLC, and identified relationships with macrophages M0, Tfh, Tregs, etc. The nomogram and the risk model constructed on the basis of MSC-related factors *POSTN, TRPA1* and *DDIT4* could facilitate the search for target molecules involved in the progression of NSCLC, and the risk model could help in the evaluation of the prognosis of NSCLC patients.

## Data Availability

The original contributions presented in the study are included in the article/Supplementary Material, further inquiries can be directed to the corresponding authors.

## Footnotes

1
http://www.ncbi.nlm.nih.gov/geo/

2
https://www.string-db.org

3
https://tcga-data.nci.nih.gov/tcga/

4
http://lce.biohpc.swmed.edu/
