# Supplementary material for: Clinical Roles of Risk Model Based on Differentially Expressed Genes in Mesenchymal Stem Cells in Prognosis and Immunity of Non-small Cell Lung Cancer
Source: Front Genet. 2022 Feb 24;13:823075. doi: 10.3389/fgene.2022.823075 (PMC8912942; doi:10.3389/fgene.2022.823075)
Supplement: Supplementary file 1 [file DataSheet1.ZIP › Raw data of Fig 1/Figure 1A.docx]

Figure 1A. Sample quality in Lung cancer tissues of GSE104636 dataset via the online GEO2R.
