# Supplementary material for: Clinical Roles of Risk Model Based on Differentially Expressed Genes in Mesenchymal Stem Cells in Prognosis and Immunity of Non-small Cell Lung Cancer
Source: Front Genet. 2022 Feb 24;13:823075. doi: 10.3389/fgene.2022.823075 (PMC8912942; doi:10.3389/fgene.2022.823075)
Supplement: Supplementary file 1 [file DataSheet1.ZIP › Raw data of Fig S3/Figure S3.docx]

The results in Figure S3 are from the lung cancer explorer (LCE) online database.
