# Supplementary material for: Clinical Roles of Risk Model Based on Differentially Expressed Genes in Mesenchymal Stem Cells in Prognosis and Immunity of Non-small Cell Lung Cancer
Source: Front Genet. 2022 Feb 24;13:823075. doi: 10.3389/fgene.2022.823075 (PMC8912942; doi:10.3389/fgene.2022.823075)
Supplement: Supplementary file 2 [file DataSheet2.docx]

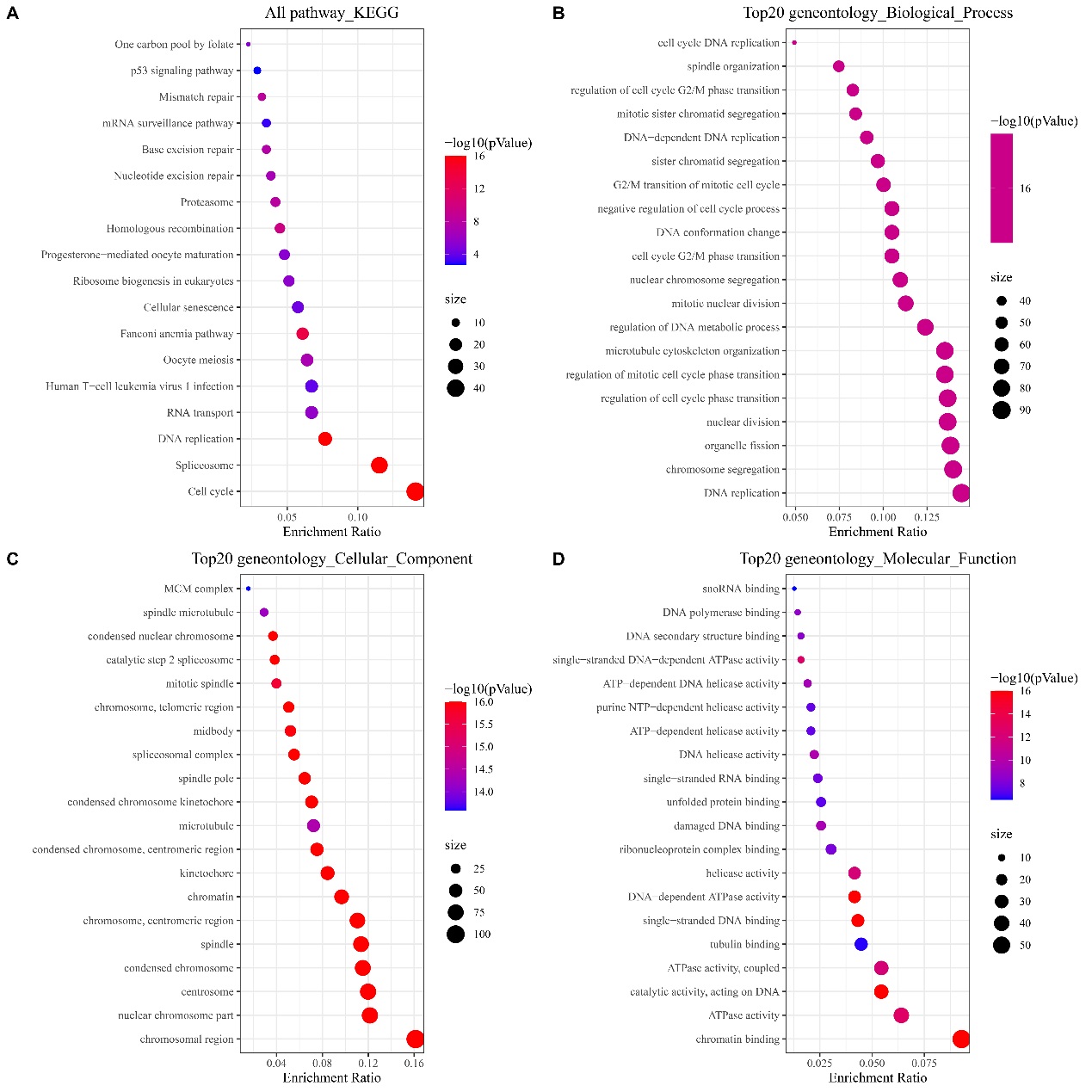


Figure S1. Functions and mechanisms associated with the DEGs of MSCs, analyzed using GO and KEGG. (A) Signaling pathways involved in MSC-related genes; (B) Biological process involved in MSC-related genes; (C) Cellular component involved in MSC-related genes; (D) Molecular function involved in MSC-related genes. DEGs, differentially expressed genes; GO, Gene ontology; KEGG, Kyoto Encyclopedia of Genes and Genomes; MSCs, Mesenchymal stem cells.


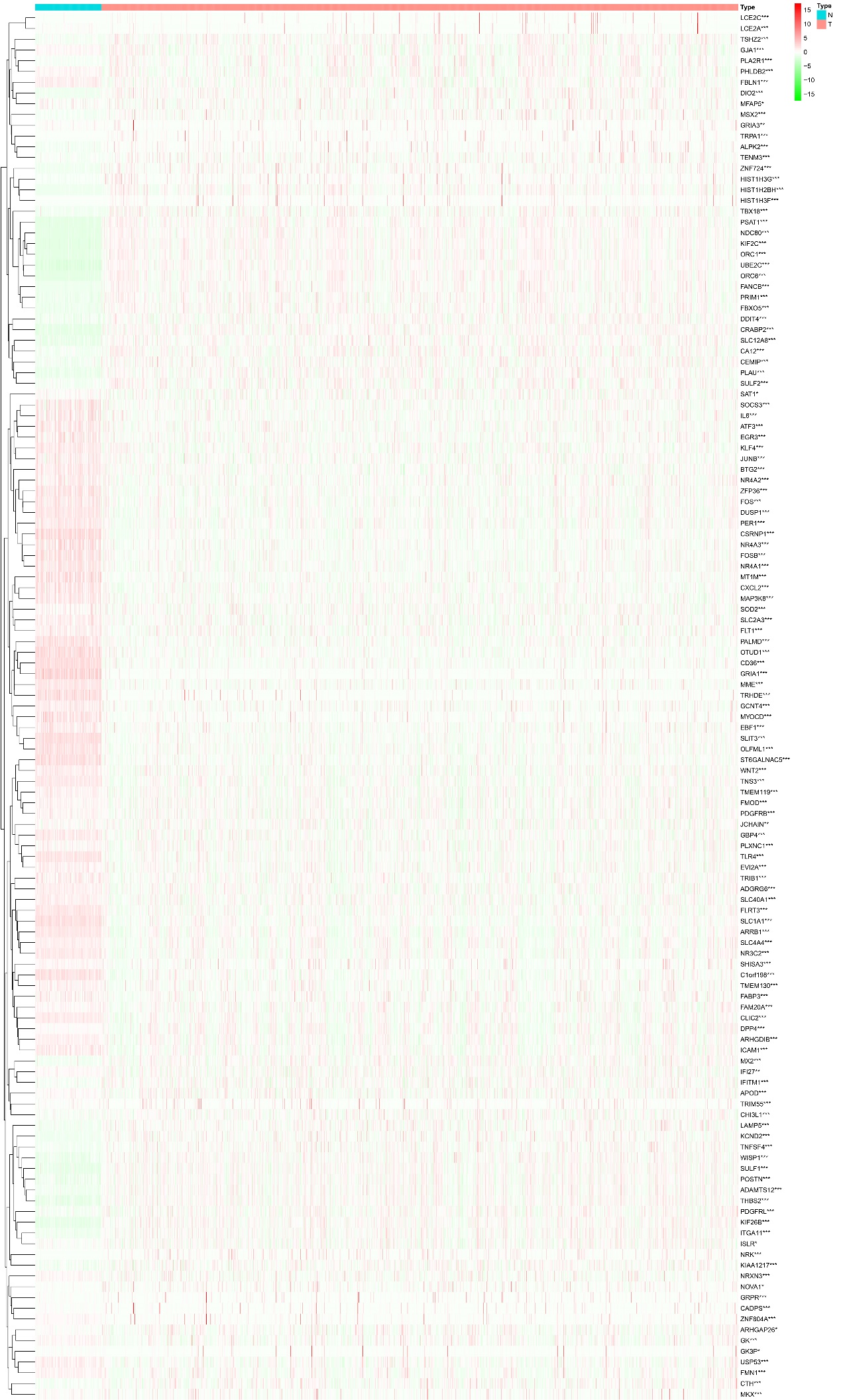


Figure S2. Heatmap of 129 MSC-related DEGs in NSCLC tissues. DEGs, differentially expressed genes; MSCs, Mesenchymal stem cells; NSCLC, Non-small-cell lung cancer.


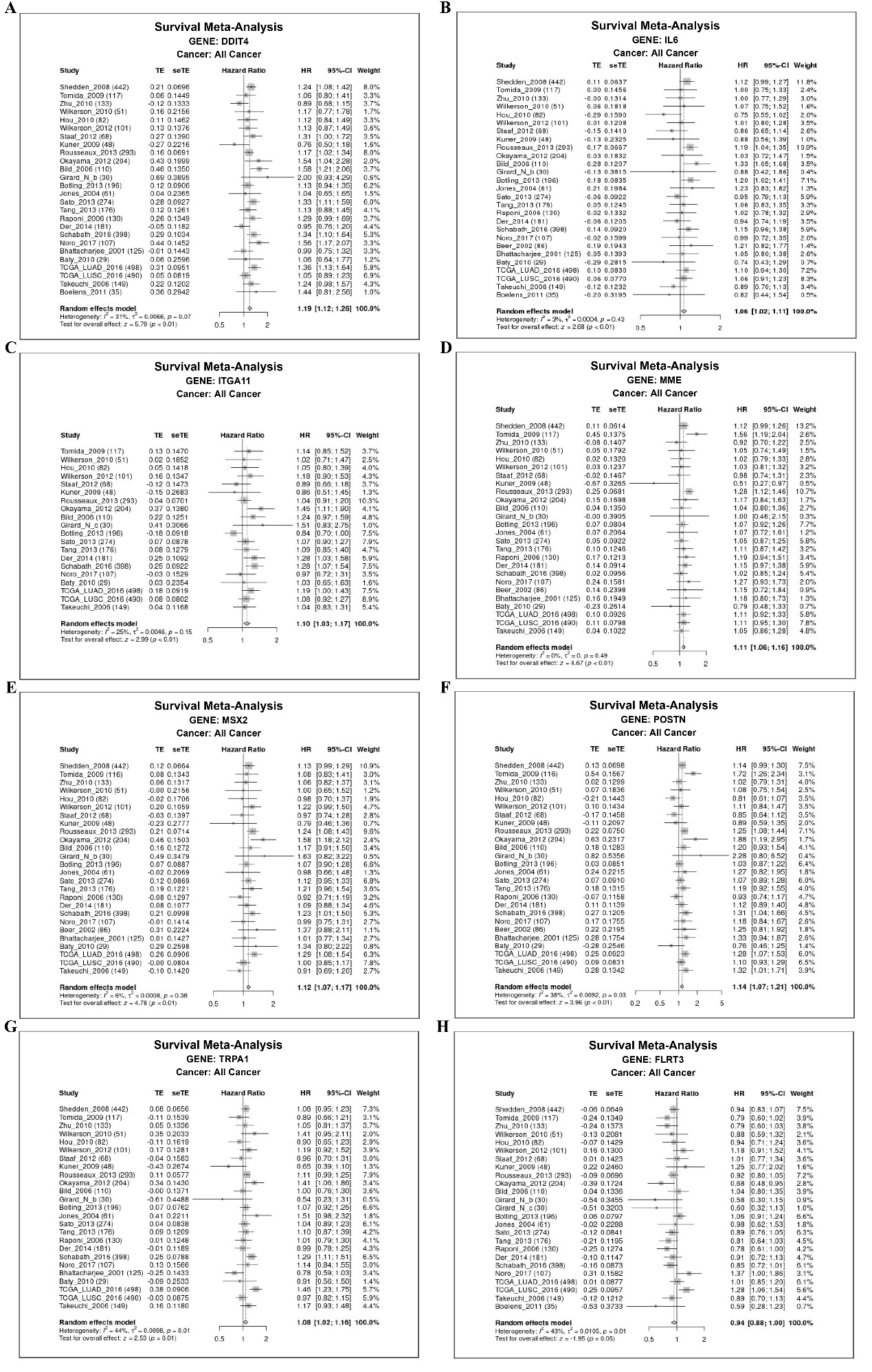


Figure S3. Potential-prognostic value of DEGs of MSCs of lung cancer patients determined using meta-analysis. (A) DDIT4; (B) IL6; (C) ITGA11; (D) MME; (E) MSX2; (F) POSTN; (G) TRPA1; (H) FLRT3. DEGs, differentially expressed genes; MSCs, Mesenchymal stem cells.


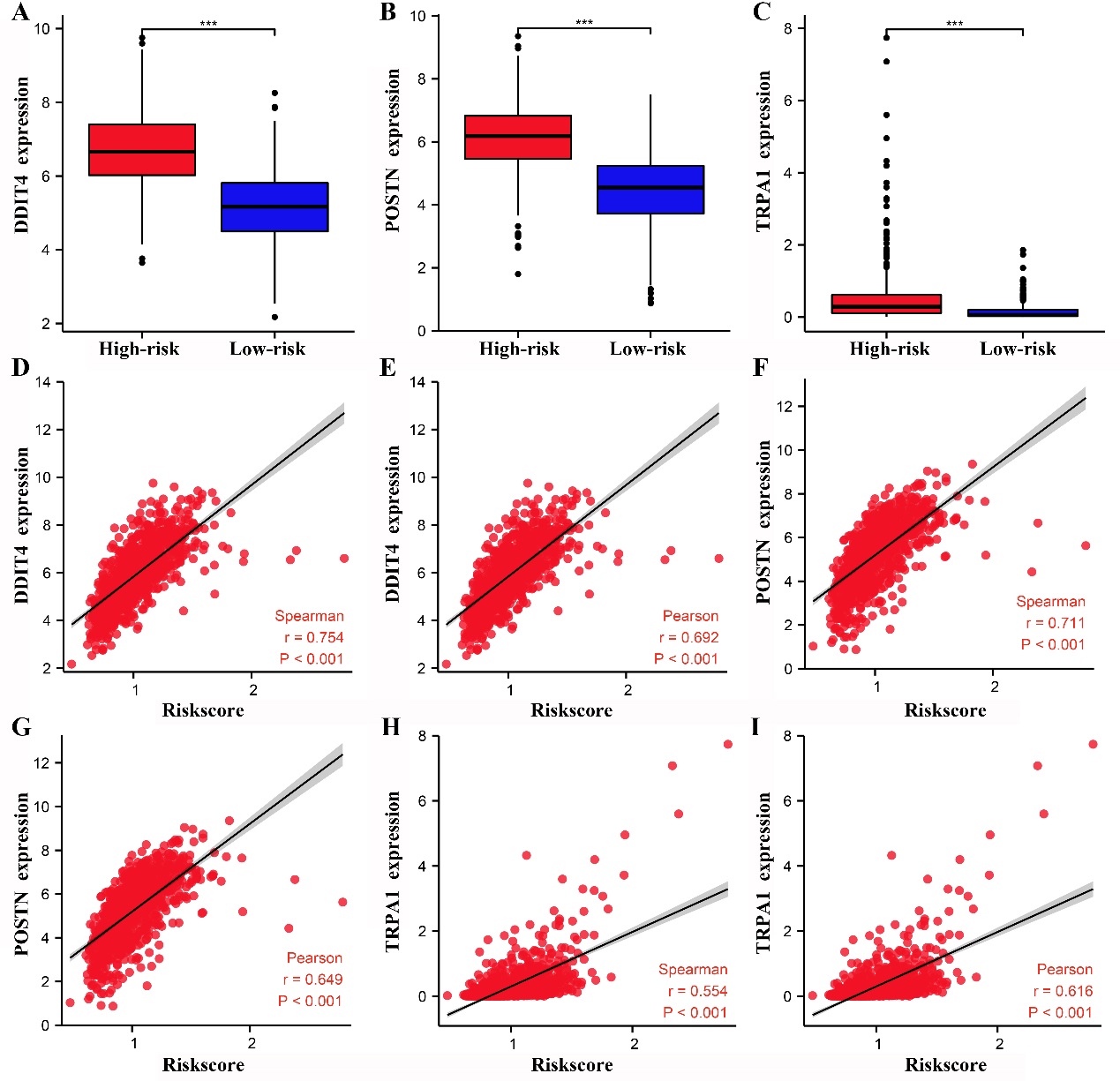


Figure S4. Prognostic risk model factors significantly correlated with risk scores. (A-C) DDIT4, POSTN, and TRPA1 expression levels in the high- and low-risk groups; (D-F) DDIT4, POSTN, and TRPA1 expression levels are positively correlated with risk scores using the Pearson correlation analysis; (G-I) DDIT4, POSTN, and TRPA1 expression levels are positively correlated with risk scores using the Spearman correlation analysis.


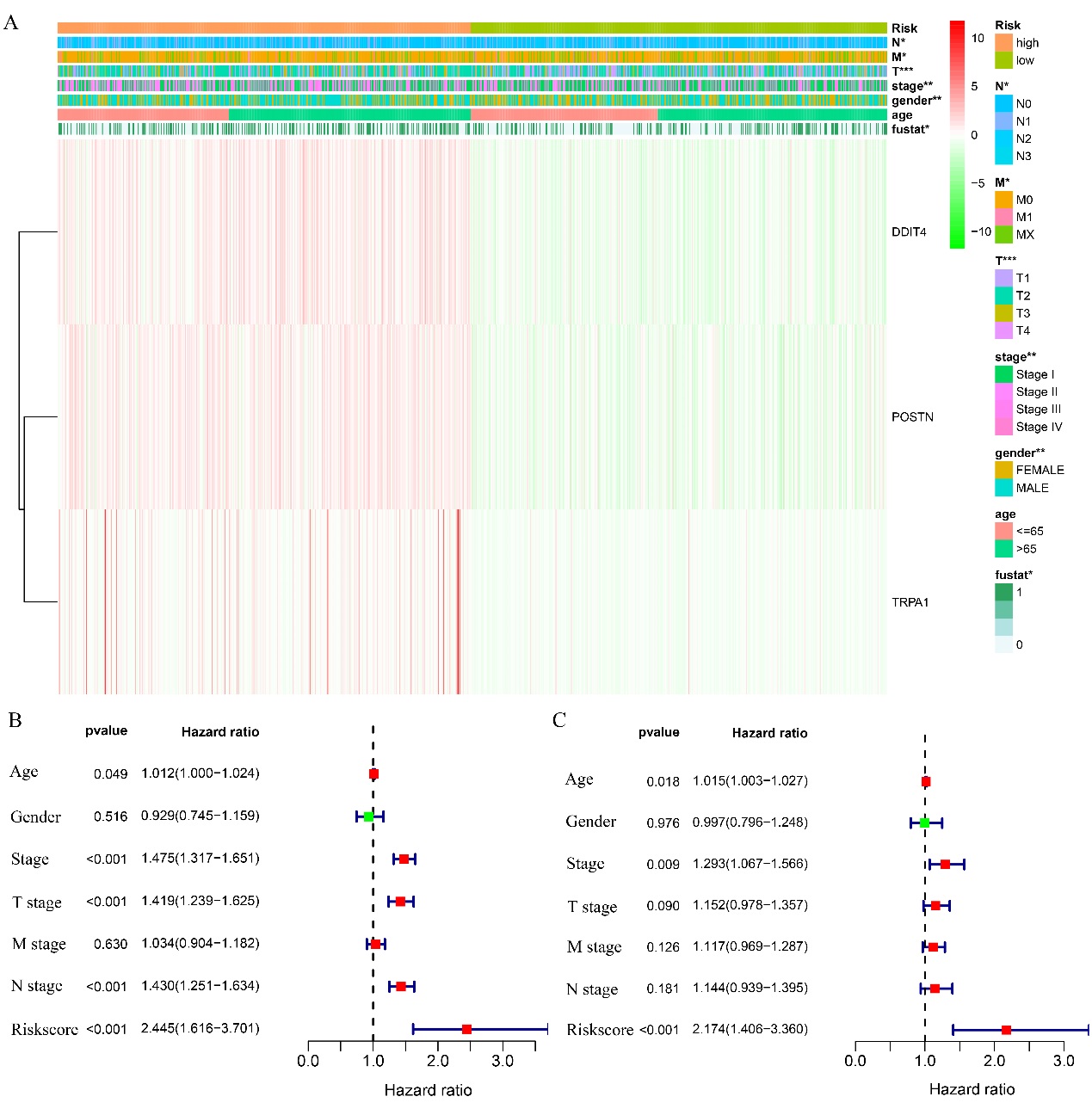


Figure S5. Risk model was associated with clinicopathological features and dismal prognosis in NSCLC patients. (A) Risk model was associated with clinicopathological features in NSCLC patients represented as a heatmap; (B–C) Risk model was associated with the dismal prognosis in NSCLC patients using the Cox regression analysis. NSCLC, Non-small-cell lung cancer.


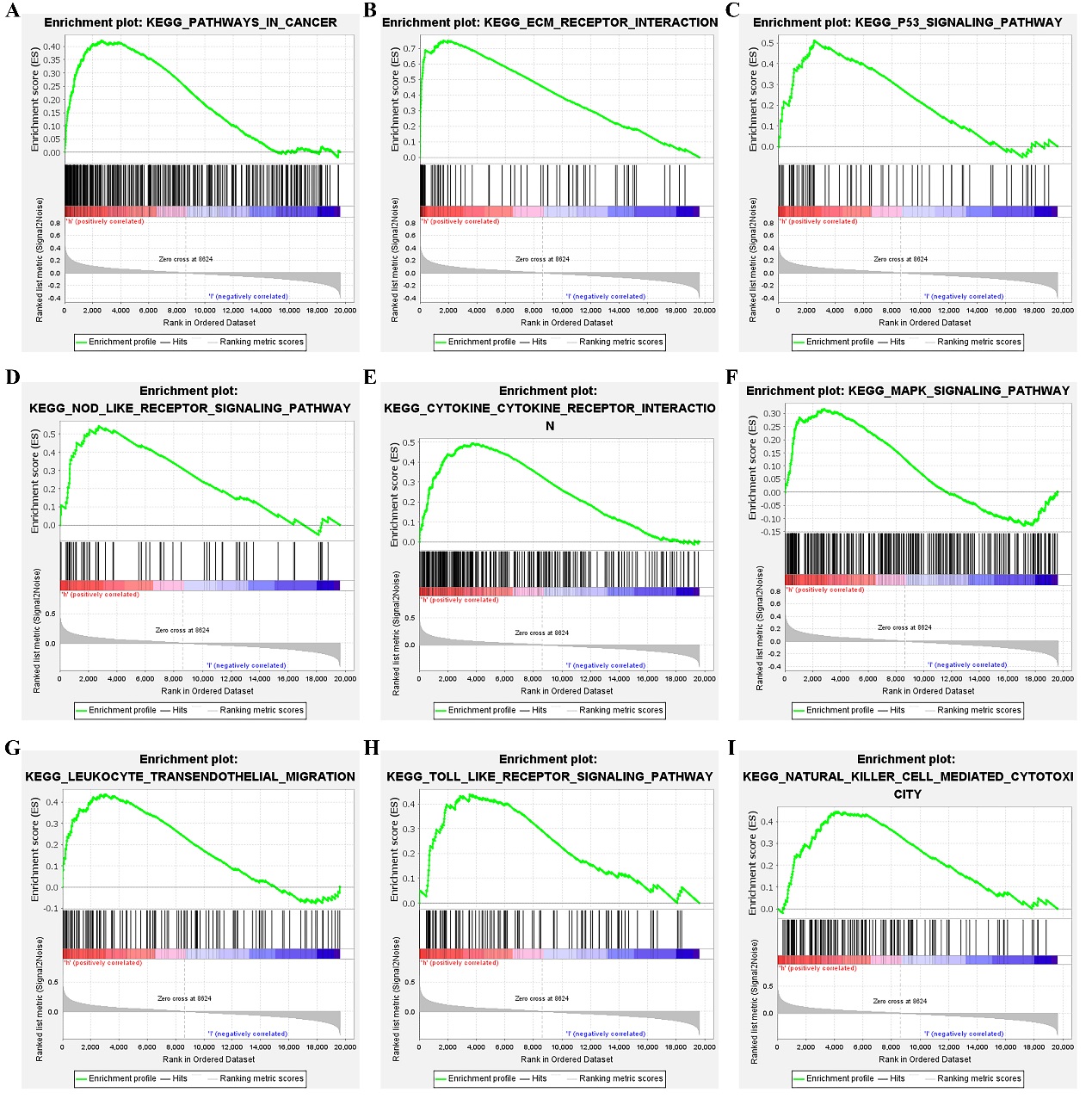


Figure S6. High-risk model might involve the signaling pathways through GSEA analysis. (A) Pathways in cancer; (B) ECM receptor interaction; (C) P53 signaling pathway; (D) NOD like receptor signaling pathway; (E) Cytokine-cytokine receptor interaction; (F) MAPK signaling pathway; (G) Leukocyte transendothelial migration; (H) Toll like receptor signaling pathway; (I) Natural killer cell mediated cytotoxicity. GSEA, Gene Set Enrichment Analysis.

Table S1. Gene expression in MSCs of lung cancer in GSE104636 dataset.

| Gene | ID | logFC | P.value |
| --- | --- | --- | --- |
| ADAMTS12 | 8111387 | -0.7957406 | 0.00073777 |
| ADAMTSL4-AS1 | 7919743 | -0.5901393 | 0.02577859 |
| ADGRG6 | 8122365 | 0.6806127 | 0.00012229 |
| ALPK2 | 8023528 | -0.6954318 | 0.00177254 |
| APOD | 8092970 | -0.9968981 | 0.01993799 |
| ARHGAP26 | 8108873 | -0.8224572 | 0.00677613 |
| ARHGDIB | 7961532 | 0.7554772 | 0.04771755 |
| ARRB1 | 7950473 | -0.8083741 | 0.0019746 |
| ASPN | 8162394 | -1.7595783 | 0.0011956 |
| ATF3 | 7909610 | -1.1708658 | 0.01072638 |
| BHLHE40 | 8077441 | -1.1479002 | 0.00274505 |
| BOC | 8081686 | -0.6092273 | 0.00035057 |
| BST2 | 8035304 | -0.9382333 | 0.0000775 |
| BTG2 | 7908917 | -0.7245124 | 0.01248714 |
| C1orf198 | 7924996 | -0.6433469 | 0.00338218 |
| C7orf69 | 8132710 | -1.5876262 | 0.00249509 |
| CA12 | 7989501 | -0.6493089 | 0.00810267 |
| CADPS | 8088491 | 1.122999 | 0.00856643 |
| CCNL1 | 8091658 | -0.6416591 | 0.01520676 |
| CD36 | 8133876 | -1.2524269 | 0.0385796 |
| CEMIP | 7985317 | -0.6818589 | 0.00844953 |
| CHI3L1 | 7923547 | -3.3608584 | 0.0000039 |
| CLIC2 | 8176234 | -0.8354494 | 0.04489397 |
| CPXM2 | 7936835 | -1.66008 | 0.00012005 |
| CRABP2 | 7921099 | -1.1926242 | 0.00294766 |
| CSRNP1 | 8086330 | -0.7184977 | 0.02201323 |
| CTH | 7902290 | -0.8491022 | 0.00600494 |
| CXCL2 | 8100994 | -0.640267 | 0.02425422 |
| DDIT4 | 7928308 | -1.0258957 | 0.00564451 |
| DIO2 | 7980485 | -0.9801027 | 0.00182151 |
| DPP4 | 8056222 | -0.9462901 | 0.00598403 |
| DUSP1 | 8115831 | -0.62661 | 0.00382538 |
| DUSP5 | 7930413 | -0.7120444 | 0.01662806 |
| EBF1///PDGFRB | 8115543 | -0.9379096 | 0.00048059 |
| EGR3 | 8149720 | -0.6360751 | 0.02057828 |
| ERRFI1 | 7912157 | -0.5881707 | 0.02796387 |
| EVI2A | 8014066 | -0.6025458 | 0.02025772 |
| FABP3 | 7914342 | -1.0205784 | 0.01495765 |
| FAM150A | 8150751 | 0.6179536 | 0.02066618 |
| FAM20A | 8017867 | -1.270668 | 0.00191954 |
| FANCB | 8171381 | 0.5952073 | 0.03206763 |
| FBLN1 | 8073775 | -1.0666727 | 0.02082635 |
| FBLN2 | 8077970 | -0.6687264 | 0.01669571 |
| FBXO5 | 8130374 | 0.7618967 | 0.04572825 |
| FLRT3 | 8065071 | 0.9165539 | 0.00121252 |
| FLT1 | 7970763 | -0.6144122 | 0.00096128 |
| FMN1 | 7987145 | 0.6631814 | 0.00284665 |
| FMOD | 7923578 | -1.277948 | 0.00934001 |
| FOS | 7975779 | -1.7473927 | 0.00249053 |
| FOSB | 8029693 | -1.7190314 | 0.00336193 |
| GBP4 | 7917561 | -0.604934 | 0.00192702 |
| GCNT4 | 8112668 | -0.7619587 | 0.00575398 |
| GJA1 | 8121749 | -0.5979431 | 0.00389183 |
| GK3P///GK | 8174103 | 0.6339701 | 0.01011946 |
| GK3P///GK | 8166632 | 0.6411279 | 0.01131676 |
| GPRC5B | 7999909 | -0.8716353 | 0.00251565 |
| GRIA1 | 8109383 | -0.8836639 | 0.04765032 |
| GRIA3 | 8169717 | -0.6753802 | 0.04854355 |
| GRPR | 8166202 | 1.4189634 | 0.0027393 |
| HAPLN1 | 8112971 | -1.2124243 | 0.01003915 |
| HIST1H2BH///HIST1H2BH | 8117426 | 0.8483701 | 0.01369835 |
| HIST1H3F | 8124437 | 0.7873906 | 0.01748819 |
| HIST1H3G | 8124440 | 0.7953944 | 0.02112042 |
| HTR2B | 8059680 | -0.902932 | 0.0070164 |
| ICAM1 | 8025601 | -0.6678259 | 0.03006222 |
| IFI27 | 7976443 | -0.6157373 | 0.00547512 |
| IFITM1 | 7937335 | -0.7340086 | 0.00295421 |
| IL6 | 8131803 | -1.5096992 | 0.00328566 |
| ISLR | 7984813 | -0.8600774 | 0.00516793 |
| ITGA11 | 7989985 | -1.7985064 | 0.00394749 |
| JCHAIN | 8100827 | -0.8199378 | 0.00310154 |
| JUNB | 8026047 | -0.8937714 | 0.00457938 |
| KCND2 | 8135705 | -0.8925007 | 0.01237806 |
| KIAA1217 | 7926679 | -0.7291321 | 0.0000456 |
| KIF26B | 7911114 | -0.773938 | 0.01753682 |
| KIF2C | 7901010 | 0.7552993 | 0.04421125 |
| KLF4 | 8163002 | -1.1610013 | 0.00333066 |
| LAMP5 | 8060940 | 1.0192406 | 0.02130347 |
| LCE2A | 7905507 | 0.9374809 | 0.01187491 |
| LCE2C | 7905503 | 1.2034977 | 0.00587778 |
| LOXL4 | 7935553 | -0.7473077 | 0.01618139 |
| MAP3K8 | 7926900 | -0.6917511 | 0.0087062 |
| MEDAG | 7968351 | -0.7111769 | 0.04448754 |
| MFAP5 | 7960919 | -1.5539297 | 0.00283175 |
| MKX | 7932733 | -0.7661388 | 0.01125013 |
| MME | 8083494 | -0.9268891 | 0.00003678 |
| MSX2 | 8110084 | -0.967472 | 0.00173424 |
| MT1M | 7995787 | 0.7479892 | 0.01795253 |
| MX2 | 8068697 | -0.7313311 | 0.00013227 |
| MYO1D | 8014115 | -1.2907437 | 0.00000616 |
| MYOCD | 8005048 | 0.8877893 | 0.00117974 |
| NDC80 | 8019857 | 0.8519394 | 0.04950027 |
| NOVA1 | 7978391 | 0.6386604 | 0.0009124 |
| NPTX1 | 8019074 | 0.9395447 | 0.03580839 |
| NR3C2 | 8103094 | 0.6115834 | 0.01390019 |
| NR4A1 | 7955589 | -1.1783741 | 0.02771516 |
| NR4A2 | 8055952 | -1.3178849 | 0.03124728 |
| NR4A3 | 8156848 | -1.0377994 | 0.0113042 |
| NRK | 8169115 | 0.722579 | 0.00885514 |
| NRXN3 | 7976012 | -0.9079486 | 0.04047923 |
| OLFML1 | 7938225 | -0.7456807 | 0.02702951 |
| ORC1 | 7916167 | 0.6012133 | 0.04153716 |
| ORC6 | 7995354 | 0.6577207 | 0.02789483 |
| OTUD1 | 7926677 | -1.0811108 | 0.00011375 |
| OXTR | 8085138 | -0.6988146 | 0.00088783 |
| PALMD | 7903227 | 0.8725153 | 0.01191767 |
| PDGFRL | 8144802 | -0.6127308 | 0.0124975 |
| PER1 | 8012349 | -0.7452532 | 0.03084758 |
| PGA4///PGA3///PGA5 | 7940421 | -0.7095586 | 0.01043237 |
| PGA4///PGA3///PGA5 | 7940441 | -0.7578829 | 0.01283836 |
| PGA4///PGA3///PGA5 | 7940431 | -0.7029839 | 0.0208197 |
| PHLDB2 | 8081590 | -0.6005181 | 0.039139 |
| PIR-FIGF///FIGF | 8171427 | -2.3863397 | 0.00023415 |
| PLA2R1 | 8056151 | -0.859831 | 0.00327989 |
| PLAU | 7928429 | 0.611912 | 0.00843639 |
| PLXNC1 | 7957570 | -1.1531242 | 0.04313971 |
| POSTN | 7971077 | -0.6495358 | 0.03489267 |
| PRIM1 | 7964271 | 0.6102368 | 0.04653138 |
| PSAT1 | 8156043 | -0.6220222 | 0.0229129 |
| RPS6KA6 | 8173825 | 0.7926748 | 0.00832049 |
| SAT1 | 8166469 | -0.6716484 | 0.02890221 |
| SEPP1 | 8111915 | -0.7338046 | 0.03299723 |
| SHISA3 | 8094870 | 0.8475746 | 0.03240066 |
| SLC12A8 | 8090214 | -0.6112222 | 0.00202601 |
| SLC1A1 | 8154135 | -0.6605351 | 0.01569989 |
| SLC2A3 | 7960865 | -0.7407322 | 0.00551274 |
| SLC40A1 | 8057677 | -0.7411834 | 0.03448021 |
| SLC4A4 | 8095585 | 0.7795953 | 0.00003768 |
| SLC7A8 | 7977933 | -1.0113111 | 0.00045062 |
| SLIT3 | 8115691 | -0.6448962 | 0.02077504 |
| SLITRK6 | 7972239 | 1.0694203 | 0.04561112 |
| SOCS3 | 8018864 | -0.7490854 | 0.01367451 |
| SOD2 | 8130556 | -0.6616241 | 0.02522388 |
| ST6GALNAC5 | 8180266 | -1.2141337 | 0.00198315 |
| ST6GALNAC5 | 7902441 | -1.1763656 | 0.00200739 |
| STK32B | 8093858 | 0.6164792 | 0.0123808 |
| SULF1 | 8146863 | -0.8132453 | 0.0037039 |
| SULF2 | 8066822 | -1.2939451 | 0.03830128 |
| TBX18 | 8127932 | -0.9855897 | 0.00044625 |
| TENM3 | 8098441 | -1.0381601 | 0.00824833 |
| TFPI2 | 8141016 | 1.455487 | 0.0027415 |
| TGFB3 | 7980316 | -0.6321403 | 0.00865761 |
| THBS2 | 8130867 | -0.8591213 | 0.02206535 |
| TLR4 | 8157524 | -0.832381 | 0.00147911 |
| TMEM119 | 7966122 | -0.6190648 | 0.013523 |
| TMEM130 | 8141228 | -0.876436 | 0.00102742 |
| TNFSF4 | 7922343 | -0.7201564 | 0.04937874 |
| TNS3 | 8139500 | -0.6064466 | 0.00027828 |
| TRHDE | 7957221 | 0.6769544 | 0.04243201 |
| TRIB1 | 8148304 | -0.7858206 | 0.00850088 |
| TRIM55 | 8146669 | 0.605662 | 0.04905463 |
| TRPA1 | 8151341 | 0.8495733 | 0.02090662 |
| TSHZ2 | 8063444 | -0.8010321 | 0.00862632 |
| TSHZ2 | 8063437 | -0.6970234 | 0.02407984 |
| UBE2C | 8063043 | 0.6457273 | 0.03248859 |
| USP53 | 8097098 | -0.7696791 | 0.00443599 |
| WISP1 | 8148435 | -0.8324452 | 0.00378044 |
| WNT2 | 8142471 | -1.3518656 | 0.00231035 |
| ZFP36 | 8028652 | -0.7823738 | 0.00155362 |
| ZNF724 | 8035838 | 0.734512 | 0.04102531 |
| ZNF804A | 8046815 | 0.7967041 | 0.00624002 |

NSCLC, Non-small-cell lung cancer; MSC, Mesenchymal stem cells.

Table S2. The functional of MSCs DEGs.

| ID | Description | p |
| --- | --- | --- |
| GO:0006260 | DNA replication | 2.25E-66 |
| GO:0007059 | chromosome segregation | 2.59E-60 |
| GO:0006261 | DNA-dependent DNA replication | 3.40E-48 |
| GO:0000280 | nuclear division | 1.06E-47 |
| GO:0000819 | sister chromatid segregation | 1.13E-47 |
| GO:0098813 | nuclear chromosome segregation | 1.36E-46 |
| GO:0140014 | mitotic nuclear division | 2.13E-46 |
| GO:0048285 | organelle fission | 7.10E-45 |
| GO:0000070 | mitotic sister chromatid segregation | 2.12E-42 |
| GO:1901990 | regulation of mitotic cell cycle phase transition | 4.00E-40 |
| GO:0044839 | cell cycle G2/M phase transition | 1.69E-38 |
| GO:1901987 | regulation of cell cycle phase transition | 3.71E-38 |
| GO:0000086 | G2/M transition of mitotic cell cycle | 1.26E-37 |
| GO:0000226 | microtubule cytoskeleton organization | 1.32E-37 |
| GO:0071103 | DNA conformation change | 4.18E-36 |
| GO:0051052 | regulation of DNA metabolic process | 5.51E-36 |
| GO:0007051 | spindle organization | 2.20E-32 |
| GO:1902749 | regulation of cell cycle G2/M phase transition | 1.56E-30 |
| GO:0044786 | cell cycle DNA replication | 2.04E-30 |
| GO:0010948 | negative regulation of cell cycle process | 2.89E-30 |
| GO:0010389 | regulation of G2/M transition of mitotic cell cycle | 1.89E-29 |
| GO:0000723 | telomere maintenance | 3.82E-29 |
| GO:0051983 | regulation of chromosome segregation | 1.91E-28 |
| GO:0000375 | RNA splicing, via transesterification reactions | 2.88E-28 |
| GO:0034502 | protein localization to chromosome | 3.92E-28 |
| GO:0000075 | cell cycle checkpoint | 4.44E-28 |
| GO:0045930 | negative regulation of mitotic cell cycle | 6.00E-28 |
| GO:0000377 | RNA splicing, via transesterification reactions  with bulged adenosine as nucleophile | 9.94E-28 |
| GO:0000398 | mRNA splicing, via spliceosome | 9.94E-28 |
| GO:0000082 | G1/S transition of mitotic cell cycle | 1.12E-27 |
| GO:0032200 | telomere organization | 1.78E-27 |
| GO:0044843 | cell cycle G1/S phase transition | 5.82E-27 |
| GO:1902850 | microtubule cytoskeleton organization involved in mitosis | 7.90E-27 |
| GO:0033260 | nuclear DNA replication | 5.76E-26 |
| GO:0007093 | mitotic cell cycle checkpoint | 8.99E-26 |
| GO:0006397 | mRNA processing | 1.15E-25 |
| GO:0006310 | DNA recombination | 2.25E-25 |
| GO:0008380 | RNA splicing | 1.30E-24 |
| GO:0007088 | regulation of mitotic nuclear division | 2.33E-24 |
| GO:0051054 | positive regulation of DNA metabolic process | 1.03E-23 |
| GO:1901988 | negative regulation of cell cycle phase transition | 1.19E-23 |
| GO:0033044 | regulation of chromosome organization | 1.54E-23 |
| GO:1901991 | negative regulation of mitotic cell cycle phase transition | 1.77E-23 |
| GO:0007052 | mitotic spindle organization | 3.27E-23 |
| GO:0071897 | DNA biosynthetic process | 8.21E-23 |
| GO:0006323 | DNA packaging | 1.27E-22 |
| GO:0006403 | RNA localization | 2.96E-22 |
| GO:0006275 | regulation of DNA replication | 4.00E-22 |
| GO:1903311 | regulation of mRNA metabolic process | 4.85E-22 |
| GO:0051783 | regulation of nuclear division | 6.02E-22 |
| GO:0033045 | regulation of sister chromatid segregation | 1.36E-21 |
| GO:0090068 | positive regulation of cell cycle process | 1.81E-21 |
| GO:0007098 | centrosome cycle | 9.89E-21 |
| GO:0045787 | positive regulation of cell cycle | 2.10E-20 |
| GO:0006302 | double-strand break repair | 3.12E-20 |
| GO:0032392 | DNA geometric change | 5.20E-20 |
| GO:0031023 | microtubule organizing center organization | 1.71E-19 |
| GO:0006270 | DNA replication initiation | 3.03E-19 |
| GO:0090329 | regulation of DNA-dependent DNA replication | 1.89E-18 |
| GO:0034508 | centromere complex assembly | 1.90E-18 |
| GO:0031570 | DNA integrity checkpoint | 2.07E-18 |
| GO:0071824 | protein-DNA complex subunit organization | 4.72E-18 |
| GO:0072331 | signal transduction by p53 class mediator | 5.14E-18 |
| GO:0043044 | ATP-dependent chromatin remodeling | 5.67E-18 |
| GO:0071459 | protein localization to chromosome, centromeric region | 5.77E-18 |
| GO:0006338 | chromatin remodeling | 6.77E-18 |
| GO:0031055 | chromatin remodeling at centromere | 1.44E-17 |
| GO:0051321 | meiotic cell cycle | 1.81E-17 |
| GO:0031145 | anaphase-promoting complex-dependent catabolic process | 2.25E-17 |
| GO:0007091 | metaphase/anaphase transition of mitotic cell cycle | 2.43E-17 |
| GO:0010965 | regulation of mitotic sister chromatid separation | 2.43E-17 |
| GO:0022613 | ribonucleoprotein complex biogenesis | 3.46E-17 |
| GO:0034080 | CENP-A containing nucleosome assembly | 3.72E-17 |
| GO:0061641 | CENP-A containing chromatin organization | 3.72E-17 |
| GO:0032201 | telomere maintenance via semi-conservative replication | 4.03E-17 |
| GO:0043486 | histone exchange | 5.65E-17 |
| GO:0065004 | protein-DNA complex assembly | 5.95E-17 |
| GO:0044784 | metaphase/anaphase transition of cell cycle | 6.58E-17 |
| GO:0051306 | mitotic sister chromatid separation | 6.58E-17 |
| GO:0033047 | regulation of mitotic sister chromatid segregation | 1.34E-16 |
| GO:0008608 | attachment of spindle microtubules to kinetochore | 1.97E-16 |
| GO:0034660 | ncRNA metabolic process | 2.34E-16 |
| GO:1905818 | regulation of chromosome separation | 2.66E-16 |
| GO:0051225 | spindle assembly | 3.70E-16 |
| GO:0006336 | DNA replication-independent nucleosome assembly | 4.13E-16 |
| GO:0034724 | DNA replication-independent nucleosome organization | 4.13E-16 |
| GO:0051304 | chromosome separation | 6.15E-16 |
| GO:0032508 | DNA duplex unwinding | 7.05E-16 |
| GO:0070507 | regulation of microtubule cytoskeleton organization | 8.03E-16 |
| GO:0006405 | RNA export from nucleus | 1.40E-15 |
| GO:0030071 | regulation of mitotic metaphase/anaphase transition | 2.28E-15 |
| GO:0050657 | nucleic acid transport | 2.34E-15 |
| GO:0050658 | RNA transport | 2.34E-15 |
| GO:1902750 | negative regulation of cell cycle G2/M phase transition | 2.56E-15 |
| GO:2000278 | regulation of DNA biosynthetic process | 2.60E-15 |
| GO:2001251 | negative regulation of chromosome organization | 2.72E-15 |
| GO:0051236 | establishment of RNA localization | 3.92E-15 |
| GO:2000573 | positive regulation of DNA biosynthetic process | 4.16E-15 |
| GO:0000724 | double-strand break repair via homologous recombination | 4.24E-15 |
| GO:0034501 | protein localization to kinetochore | 5.08E-15 |
| GO:0000725 | recombinational repair | 5.38E-15 |
| GO:1902099 | regulation of metaphase/anaphase transition of cell cycle | 5.84E-15 |
| GO:0051169 | nuclear transport | 6.45E-15 |
| GO:0031497 | chromatin assembly | 1.27E-14 |
| GO:0051310 | metaphase plate congression | 1.52E-14 |
| GO:0051168 | nuclear export | 1.55E-14 |
| GO:0034728 | nucleosome organization | 1.57E-14 |
| GO:0010833 | telomere maintenance via telomere lengthening | 2.10E-14 |
| GO:0006333 | chromatin assembly or disassembly | 2.16E-14 |
| GO:0006913 | nucleocytoplasmic transport | 2.18E-14 |
| GO:0036297 | interstrand cross-link repair | 3.28E-14 |
| GO:1903046 | meiotic cell cycle process | 6.18E-14 |
| GO:0140013 | meiotic nuclear division | 8.41E-14 |
| GO:0032886 | regulation of microtubule-based process | 9.34E-14 |
| GO:0010972 | negative regulation of G2/M transition of mitotic cell cycle | 1.11E-13 |
| GO:0000077 | DNA damage checkpoint | 1.12E-13 |
| GO:0042254 | ribosome biogenesis | 1.45E-13 |
| GO:0006334 | nucleosome assembly | 1.63E-13 |
| GO:0016072 | rRNA metabolic process | 1.66E-13 |
| GO:0000083 | regulation of transcription involved in G1/S  transition of mitotic cell cycle | 1.79E-13 |
| GO:0007080 | mitotic metaphase plate congression | 2.02E-13 |
| GO:0071426 | ribonucleoprotein complex export from nucleus | 2.18E-13 |
| GO:0071166 | ribonucleoprotein complex localization | 2.69E-13 |
| GO:0006401 | RNA catabolic process | 2.83E-13 |
| GO:0051383 | kinetochore organization | 3.09E-13 |
| GO:0050000 | chromosome localization | 3.41E-13 |
| GO:0051303 | establishment of chromosome localization | 3.41E-13 |
| GO:0044774 | mitotic DNA integrity checkpoint | 3.96E-13 |
| GO:0006611 | protein export from nucleus | 4.78E-13 |
| GO:0043487 | regulation of RNA stability | 4.78E-13 |
| GO:0007062 | sister chromatid cohesion | 5.07E-13 |
| GO:0033046 | negative regulation of sister chromatid segregation | 5.90E-13 |
| GO:0051298 | centrosome duplication | 8.75E-13 |
| GO:0051985 | negative regulation of chromosome segregation | 9.44E-13 |
| GO:0045841 | negative regulation of mitotic metaphase/  anaphase transition | 9.87E-13 |
| GO:2000816 | negative regulation of mitotic sister chromatid  separation | 9.87E-13 |
| GO:0045839 | negative regulation of mitotic nuclear division | 1.11E-12 |
| GO:0060249 | anatomical structure homeostasis | 1.16E-12 |
| GO:0051028 | mRNA transport | 1.18E-12 |
| GO:0043488 | regulation of mRNA stability | 1.34E-12 |
| GO:0000910 | cytokinesis | 1.40E-12 |
| GO:1903312 | negative regulation of mRNA metabolic process | 1.58E-12 |
| GO:1902100 | negative regulation of metaphase/anaphase  transition of cell cycle | 1.66E-12 |
| GO:1905819 | negative regulation of chromosome separation | 1.66E-12 |
| GO:0015931 | nucleobase-containing compound transport | 1.93E-12 |
| GO:0050684 | regulation of mRNA processing | 2.62E-12 |
| GO:0034470 | ncRNA processing | 3.49E-12 |
| GO:0006364 | rRNA processing | 4.41E-12 |
| GO:0033048 | negative regulation of mitotic sister chromatid  segregation | 4.41E-12 |
| GO:0061013 | regulation of mRNA catabolic process | 4.75E-12 |
| GO:0072401 | signal transduction involved in DNA integrity checkpoint | 4.78E-12 |
| GO:0072422 | signal transduction involved in DNA damage checkpoint | 4.78E-12 |
| GO:1901796 | regulation of signal transduction by p53 class mediator | 5.48E-12 |
| GO:0072395 | signal transduction involved in cell cycle checkpoint | 6.23E-12 |
| GO:0048024 | regulation of mRNA splicing, via spliceosome | 8.07E-12 |
| GO:0007004 | telomere maintenance via telomerase | 9.73E-12 |
| GO:0007099 | centriole replication | 1.26E-11 |
| GO:0044773 | mitotic DNA damage checkpoint | 1.26E-11 |
| GO:0051784 | negative regulation of nuclear division | 1.42E-11 |
| GO:0031503 | protein-containing complex localization | 1.54E-11 |
| GO:0006406 | mRNA export from nucleus | 1.64E-11 |
| GO:0031123 | RNA 3'-end processing | 1.64E-11 |
| GO:0071427 | mRNA-containing ribonucleoprotein complex  export from nucleus | 1.64E-11 |
| GO:0070200 | establishment of protein localization to telomere | 1.76E-11 |
| GO:1904356 | regulation of telomere maintenance via  telomere lengthening | 1.93E-11 |
| GO:0032204 | regulation of telomere maintenance | 2.18E-11 |
| GO:0031124 | mRNA 3'-end processing | 2.47E-11 |
| GO:0046605 | regulation of centrosome cycle | 2.70E-11 |
| GO:2001252 | positive regulation of chromosome organization | 4.15E-11 |
| GO:0032465 | regulation of cytokinesis | 5.53E-11 |
| GO:0006278 | RNA-dependent DNA biosynthetic process | 6.00E-11 |
| GO:0006402 | mRNA catabolic process | 6.76E-11 |
| GO:0098534 | centriole assembly | 7.91E-11 |
| GO:1903405 | protein localization to nuclear body | 9.36E-11 |
| GO:1904851 | positive regulation of establishment of protein  localization to telomere | 9.36E-11 |
| GO:1904867 | protein localization to Cajal body | 9.36E-11 |
| GO:0051984 | positive regulation of chromosome segregation | 1.51E-10 |
| GO:0070199 | establishment of protein localization to chromosome | 1.51E-10 |
| GO:0007094 | mitotic spindle assembly checkpoint | 1.53E-10 |
| GO:0031577 | spindle checkpoint | 1.53E-10 |
| GO:0071173 | spindle assembly checkpoint | 1.53E-10 |
| GO:0071174 | mitotic spindle checkpoint | 1.53E-10 |
| GO:0045931 | positive regulation of mitotic cell cycle | 1.54E-10 |
| GO:0000281 | mitotic cytokinesis | 1.65E-10 |
| GO:0042770 | signal transduction in response to DNA damage | 1.67E-10 |
| GO:0090307 | mitotic spindle assembly | 1.73E-10 |
| GO:0048025 | negative regulation of mRNA splicing, via spliceosome | 1.84E-10 |
| GO:0090670 | RNA localization to Cajal body | 1.84E-10 |
| GO:0090671 | telomerase RNA localization to Cajal body | 1.84E-10 |
| GO:0090672 | telomerase RNA localization | 1.84E-10 |
| GO:0090685 | RNA localization to nucleus | 1.84E-10 |
| GO:0010639 | negative regulation of organelle organization | 2.28E-10 |
| GO:0032210 | regulation of telomere maintenance via telomerase | 2.34E-10 |
| GO:0006282 | regulation of DNA repair | 2.94E-10 |
| GO:1904874 | positive regulation of telomerase RNA  localization to Cajal body | 3.18E-10 |
| GO:0000076 | DNA replication checkpoint | 3.33E-10 |
| GO:0070203 | regulation of establishment of protein localization to telomere | 3.33E-10 |
| GO:0061640 | cytoskeleton-dependent cytokinesis | 3.72E-10 |
| GO:0030261 | chromosome condensation | 3.76E-10 |
| GO:0090305 | nucleic acid phosphodiester bond hydrolysis | 3.76E-10 |
| GO:2001020 | regulation of response to DNA damage stimulus | 4.86E-10 |
| GO:0043484 | regulation of RNA splicing | 4.90E-10 |
| GO:0045005 | DNA-dependent DNA replication maintenance of fidelity | 5.72E-10 |
| GO:0097711 | ciliary basal body-plasma membrane docking | 6.57E-10 |
| GO:0022616 | DNA strand elongation | 7.04E-10 |
| GO:0050686 | negative regulation of mRNA processing | 7.17E-10 |
| GO:0051988 | regulation of attachment of spindle microtubules  to kinetochore | 9.67E-10 |
| GO:0070202 | regulation of establishment of protein localization  to chromosome | 9.67E-10 |
| GO:1904816 | positive regulation of protein localization to  chromosome, telomeric region | 9.67E-10 |
| GO:1990173 | protein localization to nucleoplasm | 9.67E-10 |
| GO:0070198 | protein localization to chromosome, telomeric region | 1.14E-09 |
| GO:0045740 | positive regulation of DNA replication | 1.26E-09 |
| GO:0032206 | positive regulation of telomere maintenance | 1.46E-09 |
| GO:0031297 | replication fork processing | 1.78E-09 |
| GO:0140053 | mitochondrial gene expression | 2.02E-09 |
| GO:0051302 | regulation of cell division | 2.43E-09 |
| GO:1904358 | positive regulation of telomere maintenance  via telomere lengthening | 2.62E-09 |
| GO:1904872 | regulation of telomerase RNA localization to  Cajal body | 2.81E-09 |
| GO:0031571 | mitotic G1 DNA damage checkpoint | 3.30E-09 |
| GO:0044819 | mitotic G1/S transition checkpoint | 3.30E-09 |
| GO:0033119 | negative regulation of RNA splicing | 3.34E-09 |
| GO:0042769 | DNA damage response, detection of DNA damage | 3.71E-09 |
| GO:0044783 | G1 DNA damage checkpoint | 4.17E-09 |
| GO:0000018 | regulation of DNA recombination | 4.32E-09 |
| GO:1904814 | regulation of protein localization to chromosome,  telomeric region | 5.51E-09 |
| GO:0006977 | DNA damage response, signal transduction by p53  class mediator resulting in cell cycle arrest | 5.57E-09 |
| GO:0072431 | signal transduction involved in mitotic G1 DNA  damage checkpoint | 7.15E-09 |
| GO:1902400 | intracellular signal transduction involved in G1  DNA damage checkpoint | 7.15E-09 |
| GO:0010824 | regulation of centrosome duplication | 7.18E-09 |
| GO:0007076 | mitotic chromosome condensation | 1.14E-08 |
| GO:0072413 | signal transduction involved in mitotic cell  cycle checkpoint | 1.16E-08 |
| GO:1902402 | signal transduction involved in mitotic DNA  damage checkpoint | 1.16E-08 |
| GO:1902403 | signal transduction involved in mitotic DNA  integrity checkpoint | 1.16E-08 |
| GO:0007050 | cell cycle arrest | 1.16E-08 |
| GO:0032212 | positive regulation of telomere maintenance  via telomerase | 1.26E-08 |
| GO:0006353 | DNA-templated transcription, termination | 1.26E-08 |
| GO:0051053 | negative regulation of DNA metabolic process | 2.20E-08 |
| GO:0051382 | kinetochore assembly | 2.22E-08 |
| GO:2000779 | regulation of double-strand break repair | 2.33E-08 |
| GO:0030330 | DNA damage response, signal transduction by  p53 class mediator | 2.80E-08 |
| GO:0010212 | response to ionizing radiation | 3.21E-08 |
| GO:0006297 | nucleotide-excision repair, DNA gap filling | 4.03E-08 |
| GO:1901989 | positive regulation of cell cycle phase transition | 4.18E-08 |
| GO:0006369 | termination of RNA polymerase II transcription | 6.04E-08 |
| GO:0032543 | mitochondrial translation | 7.37E-08 |
| GO:0070317 | negative regulation of G0 to G1 transition | 1.11E-07 |
| GO:0090224 | regulation of spindle organization | 1.11E-07 |
| GO:0046599 | regulation of centriole replication | 1.19E-07 |
| GO:0071158 | positive regulation of cell cycle arrest | 1.45E-07 |
| GO:1901992 | positive regulation of mitotic cell cycle  phase transition | 1.45E-07 |
| GO:1902036 | regulation of hematopoietic stem cell  differentiation | 1.71E-07 |
| GO:0060218 | hematopoietic stem cell differentiation | 1.72E-07 |
| GO:0006298 | mismatch repair | 2.04E-07 |
| GO:0071156 | regulation of cell cycle arrest | 2.13E-07 |
| GO:1900182 | positive regulation of protein localization to nucleus | 2.44E-07 |
| GO:2000045 | regulation of G1/S transition of mitotic cell cycle | 2.76E-07 |
| GO:0000380 | alternative mRNA splicing, via spliceosome | 2.81E-07 |
| GO:0040001 | establishment of mitotic spindle localization | 2.91E-07 |
| GO:0008156 | negative regulation of DNA replication | 2.95E-07 |
| GO:0051973 | positive regulation of telomerase activity | 2.95E-07 |
| GO:2000104 | negative regulation of DNA-dependent DNA replication | 3.00E-07 |
| GO:0070316 | regulation of G0 to G1 transition | 3.14E-07 |
| GO:0034504 | protein localization to nucleus | 3.22E-07 |
| GO:2000134 | negative regulation of G1/S transition of mitotic cell cycle | 3.30E-07 |
| GO:0007127 | meiosis I | 3.80E-07 |
| GO:0060236 | regulation of mitotic spindle organization | 3.92E-07 |
| GO:0045132 | meiotic chromosome segregation | 4.83E-07 |
| GO:0006301 | postreplication repair | 5.05E-07 |
| GO:0045023 | G0 to G1 transition | 5.05E-07 |
| GO:0006289 | nucleotide-excision repair | 5.07E-07 |
| GO:0140056 | organelle localization by membrane tethering | 5.11E-07 |
| GO:0016579 | protein deubiquitination | 5.15E-07 |
| GO:0007143 | female meiotic nuclear division | 5.66E-07 |
| GO:0061982 | meiosis I cell cycle process | 5.96E-07 |
| GO:0051972 | regulation of telomerase activity | 6.35E-07 |
| GO:0098781 | ncRNA transcription | 6.65E-07 |
| GO:1902807 | negative regulation of cell cycle G1/S phase transition | 6.76E-07 |
| GO:1900180 | regulation of protein localization to nucleus | 6.89E-07 |
| GO:0031146 | SCF-dependent proteasomal ubiquitin-dependent  protein catabolic process | 6.90E-07 |
| GO:2000105 | positive regulation of DNA-dependent DNA replication | 7.28E-07 |
| GO:0002478 | antigen processing and presentation of exogenous  peptide antigen | 8.40E-07 |
| GO:0000731 | DNA synthesis involved in DNA repair | 9.84E-07 |
| GO:0019884 | antigen processing and presentation of exogenous antigen | 1.02E-06 |
| GO:0000079 | regulation of cyclin-dependent protein serine/  threonine kinase activity | 1.06E-06 |
| GO:0010569 | regulation of double-strand break repair via  homologous recombination | 1.11E-06 |
| GO:0051653 | spindle localization | 1.11E-06 |
| GO:0022406 | membrane docking | 1.33E-06 |
| GO:0070646 | protein modification by small protein removal | 1.34E-06 |
| GO:0006409 | tRNA export from nucleus | 1.39E-06 |
| GO:0051031 | tRNA transport | 1.39E-06 |
| GO:0071431 | tRNA-containing ribonucleoprotein complex  export from nucleus | 1.39E-06 |
| GO:1901532 | regulation of hematopoietic progenitor cell  differentiation | 1.43E-06 |
| GO:1902806 | regulation of cell cycle G1/S phase transition | 1.47E-06 |
| GO:0070125 | mitochondrial translational elongation | 1.65E-06 |
| GO:0043161 | proteasome-mediated ubiquitin-dependent  protein catabolic process | 1.81E-06 |
| GO:1904029 | regulation of cyclin-dependent protein  kinase activity | 1.90E-06 |
| GO:0070126 | mitochondrial translational termination | 2.19E-06 |
| GO:0046653 | tetrahydrofolate metabolic process | 2.22E-06 |
| GO:1902751 | positive regulation of cell cycle G2/M phase transition | 2.72E-06 |
| GO:0048002 | antigen processing and presentation of peptide antigen | 2.97E-06 |
| GO:0051293 | establishment of spindle localization | 3.08E-06 |
| GO:0097064 | ncRNA export from nucleus | 3.08E-06 |
| GO:0071826 | ribonucleoprotein complex subunit organization | 3.44E-06 |
| GO:0071168 | protein localization to chromatin | 3.69E-06 |
| GO:0075733 | intracellular transport of virus | 3.89E-06 |
| GO:0006296 | nucleotide-excision repair, DNA incision, 5'-to lesion | 3.95E-06 |
| GO:0032467 | positive regulation of cytokinesis | 3.95E-06 |
| GO:0046606 | negative regulation of centrosome cycle | 4.32E-06 |
| GO:0016569 | covalent chromatin modification | 4.35E-06 |
| GO:0022618 | ribonucleoprotein complex assembly | 4.64E-06 |
| GO:0007063 | regulation of sister chromatid cohesion | 4.82E-06 |
| GO:0009314 | response to radiation | 4.82E-06 |
| GO:0031109 | microtubule polymerization or depolymerization | 4.89E-06 |
| GO:0009262 | deoxyribonucleotide metabolic process | 4.93E-06 |
| GO:0006284 | base-excision repair | 5.02E-06 |
| GO:0016572 | histone phosphorylation | 5.02E-06 |
| GO:0043624 | cellular protein complex disassembly | 5.32E-06 |
| GO:0010498 | proteasomal protein catabolic process | 5.37E-06 |
| GO:0016570 | histone modification | 5.77E-06 |
| GO:0033683 | nucleotide-excision repair, DNA incision | 6.32E-06 |
| GO:1902369 | negative regulation of RNA catabolic process | 6.32E-06 |
| GO:0031572 | G2 DNA damage checkpoint | 6.52E-06 |
| GO:0046794 | transport of virus | 6.57E-06 |
| GO:1904666 | regulation of ubiquitin protein ligase activity | 6.85E-06 |
| GO:0009200 | deoxyribonucleoside triphosphate metabolic process | 6.99E-06 |
| GO:0006521 | regulation of cellular amino acid metabolic process | 7.76E-06 |
| GO:0019985 | translesion synthesis | 7.90E-06 |
| GO:0006283 | transcription-coupled nucleotide-excision repair | 8.20E-06 |
| GO:0044766 | multi-organism transport | 8.20E-06 |
| GO:1902579 | multi-organism localization | 8.20E-06 |
| GO:0045739 | positive regulation of DNA repair | 9.14E-06 |
| GO:0044818 | mitotic G2/M transition checkpoint | 9.55E-06 |
| GO:0000381 | regulation of alternative mRNA splicing, via spliceosome | 9.81E-06 |
| GO:0043489 | RNA stabilization | 9.81E-06 |
| GO:0006415 | translational termination | 1.02E-05 |
| GO:0002244 | hematopoietic progenitor cell differentiation | 1.14E-05 |
| GO:0042023 | DNA endoreduplication | 1.14E-05 |
| GO:0051231 | spindle elongation | 1.14E-05 |
| GO:0051315 | attachment of mitotic spindle microtubules to kinetochore | 1.14E-05 |
| GO:0051493 | regulation of cytoskeleton organization | 1.16E-05 |
| GO:0008334 | histone mRNA metabolic process | 1.31E-05 |
| GO:0010971 | positive regulation of G2/M transition of mitotic cell cycle | 1.31E-05 |
| GO:0000726 | non-recombinational repair | 1.32E-05 |
| GO:2000736 | regulation of stem cell differentiation | 1.33E-05 |
| GO:0034629 | cellular protein-containing complex localization | 1.63E-05 |
| GO:0032984 | protein-containing complex disassembly | 2.01E-05 |
| GO:0006354 | DNA-templated transcription, elongation | 2.02E-05 |
| GO:0007100 | mitotic centrosome separation | 2.04E-05 |
| GO:0051782 | negative regulation of cell division | 2.37E-05 |
| GO:0006303 | double-strand break repair via nonhomologous  end joining | 2.44E-05 |
| GO:0045911 | positive regulation of DNA recombination | 2.80E-05 |
| GO:0048255 | mRNA stabilization | 2.80E-05 |
| GO:0006414 | translational elongation | 2.98E-05 |
| GO:0006760 | folic acid-containing compound metabolic process | 3.06E-05 |
| GO:0072698 | protein localization to microtubule cytoskeleton | 3.16E-05 |
| GO:0019882 | antigen processing and presentation | 3.37E-05 |
| GO:0000212 | meiotic spindle organization | 3.39E-05 |
| GO:0007077 | mitotic nuclear envelope disassembly | 3.39E-05 |
| GO:0010826 | negative regulation of centrosome duplication | 3.39E-05 |
| GO:0051299 | centrosome separation | 3.39E-05 |
| GO:1904668 | positive regulation of ubiquitin protein ligase activity | 3.39E-05 |
| GO:0032506 | cytokinetic process | 3.46E-05 |
| GO:0045840 | positive regulation of mitotic nuclear division | 3.77E-05 |
| GO:0000387 | spliceosomal snRNP assembly | 5.29E-05 |
| GO:0044380 | protein localization to cytoskeleton | 5.29E-05 |
| GO:0001833 | inner cell mass cell proliferation | 5.35E-05 |
| GO:0042559 | pteridine-containing compound biosynthetic process | 5.35E-05 |
| GO:0002479 | antigen processing and presentation of exogenous peptide  antigen via MHC class I, TAP-dependent | 5.83E-05 |
| GO:0009303 | rRNA transcription | 6.40E-05 |
| GO:0022411 | cellular component disassembly | 6.45E-05 |
| GO:0018205 | peptidyl-lysine modification | 6.68E-05 |
| GO:0061418 | regulation of transcription from RNA polymerase II  promoter in response to hypoxia | 7.46E-05 |
| GO:1901976 | regulation of cell cycle checkpoint | 8.02E-05 |
| GO:0019081 | viral translation | 8.08E-05 |
| GO:0090231 | regulation of spindle checkpoint | 8.08E-05 |
| GO:0090266 | regulation of mitotic cell cycle spindle assembly checkpoint | 8.08E-05 |
| GO:1903504 | regulation of mitotic spindle checkpoint | 8.08E-05 |
| GO:0042590 | antigen processing and presentation of exogenous  peptide antigen via MHC class I | 8.41E-05 |
| GO:0009411 | response to UV | 9.54E-05 |
| GO:0033238 | regulation of cellular amine metabolic process | 0.000106378 |
| GO:1902373 | negative regulation of mRNA catabolic process | 0.00010804 |
| GO:0002200 | somatic diversification of immune receptors | 0.000108156 |
| GO:0007064 | mitotic sister chromatid cohesion | 0.000110804 |
| GO:0090175 | regulation of establishment of planar polarity | 0.000115621 |
| GO:0042558 | pteridine-containing compound metabolic process | 0.00012261 |
| GO:0019080 | viral gene expression | 0.000141791 |
| GO:0000132 | establishment of mitotic spindle orientation | 0.00014338 |
| GO:0000959 | mitochondrial RNA metabolic process | 0.000149764 |
| GO:0007019 | microtubule depolymerization | 0.000149764 |
| GO:1903829 | positive regulation of cellular protein localization | 0.000155378 |
| GO:0006457 | protein folding | 0.000159939 |
| GO:0030397 | membrane disassembly | 0.000166277 |
| GO:0033262 | regulation of nuclear cell cycle DNA replication | 0.000166277 |
| GO:0051081 | nuclear envelope disassembly | 0.000166277 |
| GO:0001556 | oocyte maturation | 0.000183095 |
| GO:0031396 | regulation of protein ubiquitination | 0.000196176 |
| GO:0046822 | regulation of nucleocytoplasmic transport | 0.000197935 |
| GO:0045815 | positive regulation of gene expression, epigenetic | 0.000207079 |
| GO:0032205 | negative regulation of telomere maintenance | 0.000218663 |
| GO:0022412 | cellular process involved in reproduction in  multicellular organism | 0.000227969 |
| GO:0002562 | somatic diversification of immune receptors via  germline recombination within a single locus | 0.000228622 |
| GO:0016444 | somatic cell DNA recombination | 0.000228622 |
| GO:0051785 | positive regulation of nuclear division | 0.000228622 |
| GO:0006266 | DNA ligation | 0.000228781 |
| GO:0006978 | DNA damage response, signal transduction by p53  class mediator resulting in transcription of p21 class mediator | 0.000228781 |
| GO:0006997 | nucleus organization | 0.000258022 |
| GO:0051098 | regulation of binding | 0.000265884 |
| GO:0006188 | IMP biosynthetic process | 0.00026704 |
| GO:0009263 | deoxyribonucleotide biosynthetic process | 0.00026704 |
| GO:0046040 | IMP metabolic process | 0.00026704 |
| GO:0046607 | positive regulation of centrosome cycle | 0.00026704 |
| GO:0051255 | spindle midzone assembly | 0.00026704 |
| GO:0075522 | IRES-dependent viral translational initiation | 0.00026704 |
| GO:0031100 | animal organ regeneration | 0.000281161 |
| GO:0009394 | 2'-deoxyribonucleotide metabolic process | 0.000288268 |
| GO:1904357 | negative regulation of telomere maintenance via  telomere lengthening | 0.000288268 |
| GO:0006360 | transcription by RNA polymerase I | 0.000294794 |
| GO:0051101 | regulation of DNA binding | 0.00030477 |
| GO:0042276 | error-prone translesion synthesis | 0.000307668 |
| GO:0042772 | DNA damage response, signal transduction  resulting in transcription | 0.000307668 |
| GO:0006890 | retrograde vesicle-mediated transport, Golgi to ER | 0.000313761 |
| GO:1903320 | regulation of protein modification by small protein  conjugation or removal | 0.000345428 |
| GO:0051294 | establishment of spindle orientation | 0.00035609 |
| GO:2001022 | positive regulation of response to DNA damage stimulus | 0.00037342 |
| GO:0060071 | Wnt signaling pathway, planar cell polarity pathway | 0.000373949 |
| GO:0060968 | regulation of gene silencing | 0.000387044 |
| GO:0001736 | establishment of planar polarity | 0.000388344 |
| GO:0007164 | establishment of tissue polarity | 0.000388344 |
| GO:0006417 | regulation of translation | 0.00039105 |
| GO:0071478 | cellular response to radiation | 0.000403613 |
| GO:0001832 | blastocyst growth | 0.000405558 |
| GO:0070987 | error-free translesion synthesis | 0.000405558 |
| GO:0035404 | histone-serine phosphorylation | 0.000408 |
| GO:0070192 | chromosome organization involved in meiotic cell cycle | 0.000424832 |
| GO:0006368 | transcription elongation from RNA polymerase II promoter | 0.000431048 |
| GO:1900034 | regulation of cellular response to heat | 0.000431048 |
| GO:0019692 | deoxyribose phosphate metabolic process | 0.000435781 |
| GO:0043618 | regulation of transcription from RNA polymerase II  promoter in response to stress | 0.000443665 |
| GO:0051170 | import into nucleus | 0.00045427 |
| GO:0002474 | antigen processing and presentation of peptide  antigen via MHC class I | 0.00049346 |
| GO:0032211 | negative regulation of telomere maintenance via telomerase | 0.000525227 |
| GO:2000781 | positive regulation of double-strand break repair | 0.000528719 |
| GO:0097327 | response to antineoplastic agent | 0.00054002 |
| GO:0000209 | protein polyubiquitination | 0.000559657 |
| GO:0051656 | establishment of organelle localization | 0.000583419 |
| GO:0016445 | somatic diversification of immunoglobulins | 0.000626794 |
| GO:0001824 | blastocyst development | 0.000702235 |
| GO:0043620 | regulation of DNA-templated transcription in response  to stress | 0.000721841 |
| GO:0007292 | female gamete generation | 0.000764709 |
| GO:0090501 | RNA phosphodiester bond hydrolysis | 0.000764709 |
| GO:0042398 | cellular modified amino acid biosynthetic process | 0.000787322 |
| GO:0060271 | cilium assembly | 0.000800058 |
| GO:0042795 | snRNA transcription by RNA polymerase II | 0.000818253 |
| GO:0070498 | interleukin-1-mediated signaling pathway | 0.000831416 |
| GO:0062033 | positive regulation of mitotic sister chromatid segregation | 0.000835752 |
| GO:0045943 | positive regulation of transcription by RNA polymerase I | 0.000841716 |
| GO:0034605 | cellular response to heat | 0.000842085 |
| GO:0009141 | nucleoside triphosphate metabolic process | 0.000869012 |
| GO:0062012 | regulation of small molecule metabolic process | 0.000885894 |
| GO:0009301 | snRNA transcription | 0.000906598 |
| GO:0090263 | positive regulation of canonical Wnt signaling pathway | 0.000944087 |
| GO:0048524 | positive regulation of viral process | 0.000979693 |
| GO:0000729 | DNA double-strand break processing | 0.001044747 |
| GO:0050821 | protein stabilization | 0.001046377 |
| GO:0007131 | reciprocal meiotic recombination | 0.0010631 |
| GO:0046824 | positive regulation of nucleocytoplasmic transport | 0.001129803 |
| GO:0044106 | cellular amine metabolic process | 0.00113303 |
| GO:0072425 | signal transduction involved in G2 DNA damage checkpoint | 0.001137733 |
| GO:0051438 | regulation of ubiquitin-protein transferase activity | 0.00117999 |
| GO:0071456 | cellular response to hypoxia | 0.001226685 |
| GO:0001738 | morphogenesis of a polarized epithelium | 0.001236916 |
| GO:0035825 | homologous recombination | 0.001245482 |
| GO:1901998 | toxin transport | 0.001245482 |
| GO:0044782 | cilium organization | 0.001246949 |
| GO:0061647 | histone H3-K9 modification | 0.001340314 |
| GO:0009123 | nucleoside monophosphate metabolic process | 0.001401025 |
| GO:0006473 | protein acetylation | 0.001467178 |
| GO:0016073 | snRNA metabolic process | 0.001471881 |
| GO:0001325 | formation of extrachromosomal circular DNA | 0.001508653 |
| GO:0090656 | t-circle formation | 0.001508653 |
| GO:0090737 | telomere maintenance via telomere trimming | 0.001508653 |
| GO:0016447 | somatic recombination of immunoglobulin gene segments | 0.001517161 |
| GO:0009124 | nucleoside monophosphate biosynthetic process | 0.001547124 |
| GO:0006399 | tRNA metabolic process | 0.001554483 |
| GO:0031647 | regulation of protein stability | 0.001555345 |
| GO:0006730 | one-carbon metabolic process | 0.001556662 |
| GO:0031398 | positive regulation of protein ubiquitination | 0.001680599 |
| GO:0035567 | non-canonical Wnt signaling pathway | 0.001815207 |
| GO:0048863 | stem cell differentiation | 0.001834671 |
| GO:0009308 | amine metabolic process | 0.00184119 |
| GO:1903322 | positive regulation of protein modification by small  protein conjugation or removal | 0.00184119 |
| GO:0032781 | positive regulation of ATPase activity | 0.001925004 |
| GO:0007096 | regulation of exit from mitosis | 0.001956111 |
| GO:0018394 | peptidyl-lysine acetylation | 0.001962232 |
| GO:0036294 | cellular response to decreased oxygen levels | 0.00202248 |
| GO:0002223 | stimulatory C-type lectin receptor signaling pathway | 0.002085837 |
| GO:0060964 | regulation of gene silencing by miRNA | 0.002085837 |
| GO:0019886 | antigen processing and presentation of exogenous  peptide antigen via MHC class II | 0.002144327 |
| GO:0015949 | nucleobase-containing small molecule interconversion | 0.002232299 |
| GO:0090169 | regulation of spindle assembly | 0.002232299 |
| GO:1902115 | regulation of organelle assembly | 0.00228638 |
| GO:0097329 | response to antimetabolite | 0.002487597 |
| GO:1904355 | positive regulation of telomere capping | 0.002487597 |
| GO:0002220 | innate immune response activating cell surface  receptor signaling pathway | 0.002567082 |
| GO:0007569 | cell aging | 0.002567082 |
| GO:0016573 | histone acetylation | 0.002600673 |
| GO:0010458 | exit from mitosis | 0.002640171 |
| GO:0045737 | positive regulation of cyclin-dependent protein  serine/threonine kinase activity | 0.002640171 |
| GO:0051443 | positive regulation of ubiquitin-protein transferase activity | 0.002640171 |
| GO:0002495 | antigen processing and presentation of peptide  antigen via MHC class II | 0.002688365 |
| GO:0051781 | positive regulation of cell division | 0.002705757 |
| GO:0030177 | positive regulation of Wnt signaling pathway | 0.002737056 |
| GO:0060147 | regulation of posttranscriptional gene silencing | 0.002746091 |
| GO:0060966 | regulation of gene silencing by RNA | 0.002746091 |
| GO:0034248 | regulation of cellular amide metabolic process | 0.002752702 |
| GO:0071479 | cellular response to ionizing radiation | 0.002803816 |
| GO:0050434 | positive regulation of viral transcription | 0.002888009 |
| GO:0002504 | antigen processing and presentation of peptide or  polysaccharide antigen via MHC class II | 0.002892425 |
| GO:0048511 | rhythmic process | 0.002909469 |
| GO:0090502 | RNA phosphodiester bond hydrolysis, endonucleolytic | 0.003070496 |
| GO:0006312 | mitotic recombination | 0.003110434 |
| GO:0000245 | spliceosomal complex assembly | 0.003356496 |
| GO:0006305 | DNA alkylation | 0.003356496 |
| GO:0006306 | DNA methylation | 0.003356496 |
| GO:1903313 | positive regulation of mRNA metabolic process | 0.003356496 |
| GO:0018393 | internal peptidyl-lysine acetylation | 0.003450479 |
| GO:0071539 | protein localization to centrosome | 0.003613559 |
| GO:0043543 | protein acylation | 0.00366555 |
| GO:2000756 | regulation of peptidyl-lysine acetylation | 0.003669346 |
| GO:0007095 | mitotic G2 DNA damage checkpoint | 0.003831735 |
| GO:0009147 | pyrimidine nucleoside triphosphate metabolic process | 0.003831735 |
| GO:0032069 | regulation of nuclease activity | 0.003831735 |
| GO:0046655 | folic acid metabolic process | 0.003831735 |
| GO:0019083 | viral transcription | 0.00394205 |
| GO:0043966 | histone H3 acetylation | 0.004049462 |
| GO:0048599 | oocyte development | 0.00414946 |
| GO:1905508 | protein localization to microtubule organizing center | 0.004186097 |
| GO:0009162 | deoxyribonucleoside monophosphate metabolic process | 0.004298376 |
| GO:0032070 | regulation of deoxyribonuclease activity | 0.004298376 |
| GO:0070341 | fat cell proliferation | 0.004298376 |
| GO:0070344 | regulation of fat cell proliferation | 0.004298376 |
| GO:0070601 | centromeric sister chromatid cohesion | 0.004298376 |
| GO:1901838 | positive regulation of transcription of nucleolar large  rRNA by RNA polymerase I | 0.004298376 |
| GO:0071453 | cellular response to oxygen levels | 0.004350944 |
| GO:0006475 | internal protein amino acid acetylation | 0.004513127 |
| GO:0007281 | germ cell development | 0.004537802 |
| GO:0043902 | positive regulation of multi-organism process | 0.00457595 |
| GO:0006998 | nuclear envelope organization | 0.004648678 |
| GO:0000966 | RNA 5'-end processing | 0.004658362 |
| GO:1901673 | regulation of mitotic spindle assembly | 0.004658362 |
| GO:0051099 | positive regulation of binding | 0.004804706 |
| GO:1905330 | regulation of morphogenesis of an epithelium | 0.004804706 |
| GO:0006356 | regulation of transcription by RNA polymerase I | 0.004820517 |
| GO:0097421 | liver regeneration | 0.004820517 |
| GO:1904031 | positive regulation of cyclin-dependent protein kinase activity | 0.004820517 |
| GO:0009144 | purine nucleoside triphosphate metabolic process | 0.004828691 |
| GO:0007623 | circadian rhythm | 0.004995402 |
| GO:1903578 | regulation of ATP metabolic process | 0.005339623 |
| GO:0006370 | 7-methylguanosine mRNA capping | 0.005520266 |
| GO:0043928 | exonucleolytic nuclear-transcribed mRNA catabolic  process involved in deadenylation-dependent decay | 0.005520266 |
| GO:0034644 | cellular response to UV | 0.005530624 |
| GO:0001682 | tRNA 5'-leader removal | 0.005757268 |
| GO:0009143 | nucleoside triphosphate catabolic process | 0.005757268 |
| GO:0009151 | purine deoxyribonucleotide metabolic process | 0.005757268 |
| GO:0016446 | somatic hypermutation of immunoglobulin genes | 0.005757268 |
| GO:0043248 | proteasome assembly | 0.005757268 |
| GO:0072711 | cellular response to hydroxyurea | 0.005757268 |
| GO:0002204 | somatic recombination of immunoglobulin genes  involved in immune response | 0.005777653 |
| GO:0002208 | somatic diversification of immunoglobulins  involved in immune response | 0.005777653 |
| GO:0045143 | homologous chromosome segregation | 0.005777653 |
| GO:0045190 | isotype switching | 0.005777653 |
| GO:0007018 | microtubule-based movement | 0.005795699 |
| GO:0016925 | protein sumoylation | 0.005978331 |
| GO:0000291 | nuclear-transcribed mRNA catabolic process, exonucleolytic | 0.006288743 |
| GO:0009452 | 7-methylguanosine RNA capping | 0.006288743 |
| GO:0036260 | RNA capping | 0.006288743 |
| GO:0043967 | histone H4 acetylation | 0.006413845 |
| GO:2001021 | negative regulation of response to DNA damage stimulus | 0.006453051 |
| GO:0009116 | nucleoside metabolic process | 0.006846432 |
| GO:0042752 | regulation of circadian rhythm | 0.006846432 |
| GO:0046782 | regulation of viral transcription | 0.006989158 |
| GO:0009994 | oocyte differentiation | 0.007094497 |
| GO:0007339 | binding of sperm to zona pellucida | 0.007129286 |
| GO:0002566 | somatic diversification of immune receptors via somatic mutation | 0.007478005 |
| GO:0010457 | centriole-centriole cohesion | 0.007478005 |
| GO:0060707 | trophoblast giant cell differentiation | 0.007478005 |
| GO:0072710 | response to hydroxyurea | 0.007478005 |
| GO:1900402 | regulation of carbohydrate metabolic process by  regulation of transcription from RNA polymerase II promoter | 0.007478005 |
| GO:0006352 | DNA-templated transcription, initiation | 0.007715266 |
| GO:0097150 | neuronal stem cell population maintenance | 0.007834412 |
| GO:0046785 | microtubule polymerization | 0.008253354 |
| GO:0009127 | purine nucleoside monophosphate biosynthetic process | 0.008539323 |
| GO:0009168 | purine ribonucleoside monophosphate biosynthetic process | 0.008539323 |
| GO:0035065 | regulation of histone acetylation | 0.008615655 |
| GO:0009167 | purine ribonucleoside monophosphate metabolic process | 0.008616839 |
| GO:0043687 | post-translational protein modification | 0.008661983 |
| GO:0009126 | purine nucleoside monophosphate metabolic process | 0.008903841 |
| GO:0098687 | chromosomal region | 4.76E-71 |
| GO:0000793 | condensed chromosome | 1.43E-57 |
| GO:0000775 | chromosome, centromeric region | 1.89E-56 |
| GO:0000776 | kinetochore | 2.15E-45 |
| GO:0000779 | condensed chromosome, centromeric region | 4.47E-42 |
| GO:0000777 | condensed chromosome kinetochore | 2.34E-40 |
| GO:0005819 | spindle | 2.81E-39 |
| GO:0044454 | nuclear chromosome part | 3.44E-32 |
| GO:0005813 | centrosome | 3.90E-31 |
| GO:0000922 | spindle pole | 2.66E-26 |
| GO:0005681 | spliceosomal complex | 1.87E-19 |
| GO:0000785 | chromatin | 5.93E-19 |
| GO:0071013 | catalytic step 2 spliceosome | 2.67E-17 |
| GO:0000794 | condensed nuclear chromosome | 3.30E-17 |
| GO:0000781 | chromosome, telomeric region | 4.46E-17 |
| GO:0030496 | midbody | 1.11E-16 |
| GO:0072686 | mitotic spindle | 2.10E-16 |
| GO:0005874 | microtubule | 3.90E-15 |
| GO:0005876 | spindle microtubule | 6.09E-15 |
| GO:0042555 | MCM complex | 2.64E-14 |
| GO:0000790 | nuclear chromatin | 3.48E-12 |
| GO:0000784 | nuclear chromosome, telomeric region | 3.85E-12 |
| GO:0000940 | condensed chromosome outer kinetochore | 1.44E-11 |
| GO:0005657 | replication fork | 2.43E-11 |
| GO:0034399 | nuclear periphery | 6.42E-11 |
| GO:0016607 | nuclear speck | 6.59E-11 |
| GO:0044450 | microtubule organizing center part | 2.22E-10 |
| GO:0016363 | nuclear matrix | 6.15E-10 |
| GO:0005814 | centriole | 1.82E-09 |
| GO:0005635 | nuclear envelope | 2.95E-09 |
| GO:0005684 | U2-type spliceosomal complex | 6.12E-09 |
| GO:0046540 | U4/U6 x U5 tri-snRNP complex | 3.17E-08 |
| GO:0097526 | spliceosomal tri-snRNP complex | 3.17E-08 |
| GO:0030532 | small nuclear ribonucleoprotein complex | 3.95E-08 |
| GO:0097525 | spliceosomal snRNP complex | 6.61E-08 |
| GO:0005697 | telomerase holoenzyme complex | 1.12E-07 |
| GO:0101031 | chaperone complex | 1.12E-07 |
| GO:0051233 | spindle midzone | 1.92E-07 |
| GO:0120114 | Sm-like protein family complex | 1.96E-07 |
| GO:0000792 | heterochromatin | 2.67E-07 |
| GO:0015030 | Cajal body | 2.67E-07 |
| GO:0071007 | U2-type catalytic step 2 spliceosome | 5.33E-07 |
| GO:0071005 | U2-type precatalytic spliceosome | 5.91E-07 |
| GO:0071011 | precatalytic spliceosome | 5.91E-07 |
| GO:0005643 | nuclear pore | 7.25E-07 |
| GO:0000780 | condensed nuclear chromosome, centromeric region | 8.67E-07 |
| GO:0000502 | proteasome complex | 1.06E-06 |
| GO:1905369 | endopeptidase complex | 1.27E-06 |
| GO:0044452 | nucleolar part | 1.33E-06 |
| GO:0034709 | methylosome | 1.35E-06 |
| GO:0034708 | methyltransferase complex | 2.02E-06 |
| GO:0090734 | site of DNA damage | 2.15E-06 |
| GO:0043240 | Fanconi anaemia nuclear complex | 2.44E-06 |
| GO:0005721 | pericentric heterochromatin | 3.16E-06 |
| GO:0030894 | replisome | 3.16E-06 |
| GO:0000800 | lateral element | 4.14E-06 |
| GO:0018995 | host | 4.15E-06 |
| GO:0043657 | host cell | 4.15E-06 |
| GO:1905368 | peptidase complex | 6.58E-06 |
| GO:0044798 | nuclear transcription factor complex | 7.74E-06 |
| GO:0005759 | mitochondrial matrix | 7.92E-06 |
| GO:0044215 | other organism | 1.02E-05 |
| GO:0044216 | other organism cell | 1.02E-05 |
| GO:0044217 | other organism part | 1.02E-05 |
| GO:0031965 | nuclear membrane | 1.85E-05 |
| GO:1990023 | mitotic spindle midzone | 1.97E-05 |
| GO:0005732 | small nucleolar ribonucleoprotein complex | 2.23E-05 |
| GO:0097431 | mitotic spindle pole | 2.23E-05 |
| GO:1990752 | microtubule end | 2.23E-05 |
| GO:0090575 | RNA polymerase II transcription factor complex | 2.75E-05 |
| GO:0043596 | nuclear replication fork | 2.92E-05 |
| GO:0035145 | exon-exon junction complex | 3.22E-05 |
| GO:0008278 | cohesin complex | 3.27E-05 |
| GO:0000313 | organellar ribosome | 3.31E-05 |
| GO:0005761 | mitochondrial ribosome | 3.31E-05 |
| GO:0001650 | fibrillar center | 5.25E-05 |
| GO:0061695 | transferase complex, transferring phosphorus-containing  groups | 6.49E-05 |
| GO:0010369 | chromocenter | 7.80E-05 |
| GO:0005637 | nuclear inner membrane | 0.000102734 |
| GO:0022624 | proteasome accessory complex | 0.000106439 |
| GO:1902562 | H4 histone acetyltransferase complex | 0.000117178 |
| GO:0005689 | U12-type spliceosomal complex | 0.000137761 |
| GO:0000795 | synaptonemal complex | 0.00014316 |
| GO:0099086 | synaptonemal structure | 0.00014316 |
| GO:0035371 | microtubule plus-end | 0.000160662 |
| GO:0005667 | transcription factor complex | 0.000208524 |
| GO:0000930 | gamma-tubulin complex | 0.000297403 |
| GO:0043601 | nuclear replisome | 0.000297403 |
| GO:0000152 | nuclear ubiquitin ligase complex | 0.000297613 |
| GO:0005685 | U1 snRNP | 0.000342428 |
| GO:0035861 | site of double-strand break | 0.000353115 |
| GO:0005652 | nuclear lamina | 0.000396728 |
| GO:0005687 | U4 snRNP | 0.000396728 |
| GO:0000307 | cyclin-dependent protein kinase holoenzyme complex | 0.000419151 |
| GO:0005839 | proteasome core complex | 0.000507916 |
| GO:0000803 | sex chromosome | 0.000508651 |
| GO:0019866 | organelle inner membrane | 0.000511315 |
| GO:0000315 | organellar large ribosomal subunit | 0.000597564 |
| GO:0005762 | mitochondrial large ribosomal subunit | 0.000597564 |
| GO:0042575 | DNA polymerase complex | 0.000812993 |
| GO:0000123 | histone acetyltransferase complex | 0.000861333 |
| GO:0005720 | nuclear heterochromatin | 0.000868241 |
| GO:0005671 | Ada2/Gcn5/Ada3 transcription activator complex | 0.001468165 |
| GO:0045120 | pronucleus | 0.001468165 |
| GO:0017053 | transcriptional repressor complex | 0.001534637 |
| GO:0035097 | histone methyltransferase complex | 0.001827192 |
| GO:0031248 | protein acetyltransferase complex | 0.001835302 |
| GO:1902493 | acetyltransferase complex | 0.001835302 |
| GO:0034719 | SMN-Sm protein complex | 0.002421814 |
| GO:0090543 | Flemming body | 0.002557401 |
| GO:0044665 | MLL1/2 complex | 0.00300287 |
| GO:0071339 | MLL1 complex | 0.00300287 |
| GO:0032153 | cell division site | 0.003524841 |
| GO:0032155 | cell division site part | 0.003524841 |
| GO:0043073 | germ cell nucleus | 0.003731903 |
| GO:0005838 | proteasome regulatory particle | 0.004209829 |
| GO:0072687 | meiotic spindle | 0.004209829 |
| GO:0045171 | intercellular bridge | 0.004284882 |
| GO:0005680 | anaphase-promoting complex | 0.00545325 |
| GO:0005881 | cytoplasmic microtubule | 0.005649113 |
| GO:0032993 | protein-DNA complex | 0.005998802 |
| GO:0035770 | ribonucleoprotein granule | 0.006280791 |
| GO:0097346 | INO80-type complex | 0.006484125 |
| GO:1902554 | serine/threonine protein kinase complex | 0.007314749 |
| GO:0005840 | ribosome | 0.007397835 |
| GO:0098798 | mitochondrial protein complex | 0.00782087 |
| GO:0000428 | DNA-directed RNA polymerase complex | 0.00888959 |
| GO:0055029 | nuclear DNA-directed RNA polymerase complex | 0.00888959 |
| GO:0000178 | exosome (RNase complex) | 0.008915501 |
| GO:1905354 | exoribonuclease complex | 0.008915501 |
| GO:0001741 | XY body | 0.009280871 |
| GO:0030880 | RNA polymerase complex | 0.01088888 |
| GO:0033276 | transcription factor TFTC complex | 0.011510115 |
| GO:0035267 | NuA4 histone acetyltransferase complex | 0.011510115 |
| GO:0043189 | H4/H2A histone acetyltransferase complex | 0.011510115 |
| GO:0070461 | SAGA-type complex | 0.011874623 |
| GO:0032154 | cleavage furrow | 0.011928869 |
| GO:0000314 | organellar small ribosomal subunit | 0.013563797 |
| GO:0005763 | mitochondrial small ribosomal subunit | 0.013563797 |
| GO:0001673 | male germ cell nucleus | 0.014020603 |
| GO:0005662 | DNA replication factor A complex | 0.014020603 |
| GO:0031011 | Ino80 complex | 0.014020603 |
| GO:0033202 | DNA helicase complex | 0.014020603 |
| GO:0005875 | microtubule associated complex | 0.014932329 |
| GO:1904949 | ATPase complex | 0.014932329 |
| GO:0008094 | DNA-dependent ATPase activity | 1.78E-19 |
| GO:0003697 | single-stranded DNA binding | 8.70E-18 |
| GO:0140097 | catalytic activity, acting on DNA | 1.19E-17 |
| GO:0003682 | chromatin binding | 1.54E-17 |
| GO:0016887 | ATPase activity | 1.70E-13 |
| GO:0043142 | single-stranded DNA-dependent ATPase activity | 2.64E-13 |
| GO:0004386 | helicase activity | 9.21E-13 |
| GO:0042623 | ATPase activity, coupled | 9.21E-13 |
| GO:0003678 | DNA helicase activity | 1.49E-10 |
| GO:0003684 | damaged DNA binding | 3.14E-10 |
| GO:0004003 | ATP-dependent DNA helicase activity | 4.17E-10 |
| GO:0070182 | DNA polymerase binding | 2.20E-09 |
| GO:0000217 | DNA secondary structure binding | 3.17E-09 |
| GO:0043021 | ribonucleoprotein complex binding | 1.15E-08 |
| GO:0003727 | single-stranded RNA binding | 1.91E-08 |
| GO:0008026 | ATP-dependent helicase activity | 3.43E-08 |
| GO:0070035 | purine NTP-dependent helicase activity | 3.43E-08 |
| GO:0051082 | unfolded protein binding | 4.94E-08 |
| GO:0015631 | tubulin binding | 2.02E-07 |
| GO:0030515 | snoRNA binding | 2.76E-07 |
| GO:0042393 | histone binding | 4.55E-07 |
| GO:0000400 | four-way junction DNA binding | 7.61E-07 |
| GO:0008022 | protein C-terminus binding | 5.21E-06 |
| GO:0035173 | histone kinase activity | 5.68E-06 |
| GO:0008187 | poly-pyrimidine tract binding | 9.35E-06 |
| GO:0034061 | DNA polymerase activity | 1.56E-05 |
| GO:0042826 | histone deacetylase binding | 2.18E-05 |
| GO:0042162 | telomeric DNA binding | 2.52E-05 |
| GO:0003730 | mRNA 3'-UTR binding | 3.05E-05 |
| GO:0051539 | 4 iron, 4 sulfur cluster binding | 3.16E-05 |
| GO:0008017 | microtubule binding | 4.18E-05 |
| GO:1990825 | sequence-specific mRNA binding | 4.26E-05 |
| GO:0003887 | DNA-directed DNA polymerase activity | 8.24E-05 |
| GO:0031490 | chromatin DNA binding | 0.000113666 |
| GO:0008409 | 5'-3' exonuclease activity | 0.000207769 |
| GO:1990841 | promoter-specific chromatin binding | 0.000244942 |
| GO:0031491 | nucleosome binding | 0.000384207 |
| GO:0003725 | double-stranded RNA binding | 0.000417365 |
| GO:0016893 | endonuclease activity, active with either ribo- or  deoxyribonucleic acids and producing 5'-phosphomonoesters | 0.000489488 |
| GO:0008266 | poly(U) RNA binding | 0.000652547 |
| GO:0031492 | nucleosomal DNA binding | 0.000679516 |
| GO:0043024 | ribosomal small subunit binding | 0.000712302 |
| GO:0004527 | exonuclease activity | 0.000747366 |
| GO:0000287 | magnesium ion binding | 0.000747592 |
| GO:0036002 | pre-mRNA binding | 0.000754337 |
| GO:0004298 | threonine-type endopeptidase activity | 0.000830731 |
| GO:0070003 | threonine-type peptidase activity | 0.000830731 |
| GO:0070034 | telomerase RNA binding | 0.000830731 |
| GO:0070717 | poly-purine tract binding | 0.000830731 |
| GO:0003777 | microtubule motor activity | 0.000902943 |
| GO:0018024 | histone-lysine N-methyltransferase activity | 0.000902943 |
| GO:0016646 | oxidoreductase activity, acting on the CH-NH group  of donors, NAD or NADP as acceptor | 0.00099905 |
| GO:0051536 | iron-sulfur cluster binding | 0.001087668 |
| GO:0051540 | metal cluster binding | 0.001087668 |
| GO:0016779 | nucleotidyltransferase activity | 0.001434437 |
| GO:0003712 | transcription coregulator activity | 0.00147814 |
| GO:0004518 | nuclease activity | 0.001670229 |
| GO:0042054 | histone methyltransferase activity | 0.002818414 |
| GO:0008536 | Ran GTPase binding | 0.003243316 |
| GO:0004536 | deoxyribonuclease activity | 0.003653333 |
| GO:0001091 | RNA polymerase II basal transcription factor binding | 0.003692881 |
| GO:0008301 | DNA binding, bending | 0.004543092 |
| GO:0008327 | methyl-CpG binding | 0.004543092 |
| GO:0044389 | ubiquitin-like protein ligase binding | 0.004735866 |
| GO:0016279 | protein-lysine N-methyltransferase activity | 0.004756902 |
| GO:0004523 | RNA-DNA hybrid ribonuclease activity | 0.004915416 |
| GO:0009982 | pseudouridine synthase activity | 0.004915416 |
| GO:0016888 | endodeoxyribonuclease activity, producing 5'-  phosphomonoesters | 0.004915416 |
| GO:0046974 | histone methyltransferase activity (H3-K9 specific) | 0.004915416 |
| GO:0097617 | annealing activity | 0.004915416 |
| GO:0098505 | G-rich strand telomeric DNA binding | 0.004915416 |
| GO:0140098 | catalytic activity, acting on RNA | 0.005033511 |
| GO:0016891 | endoribonuclease activity, producing 5'-phosphomonoesters | 0.005120917 |
| GO:0016796 | exonuclease activity, active with either ribo- or  deoxyribonucleic acids and producing 5'-phosphomonoesters | 0.005226705 |
| GO:0016278 | lysine N-methyltransferase activity | 0.005242119 |
| GO:0001094 | TFIID-class transcription factor complex binding | 0.006575298 |
| GO:0032404 | mismatch repair complex binding | 0.006575298 |
| GO:0043047 | single-stranded telomeric DNA binding | 0.006575298 |
| GO:0016645 | oxidoreductase activity, acting on the CH-NH group of donors | 0.00661808 |

MSC, Mesenchymal stem cells; DEGs, differentially expressed genes.
